# Modulation of thalamocortical oscillations by TRIP8b, an auxiliary subunit for HCN channels

**DOI:** 10.1007/s00429-017-1559-z

**Published:** 2017-11-22

**Authors:** Mehrnoush Zobeiri, Rahul Chaudhary, Maia Datunashvili, Robert J. Heuermann, Annika Lüttjohann, Venu Narayanan, Sabine Balfanz, Patrick Meuth, Dane M. Chetkovich, Hans-Christian Pape, Arnd Baumann, Gilles van Luijtelaar, Thomas Budde

**Affiliations:** 1 Institut für Physiologie I, Westfälische Wilhelms-Universität, 48149 Münster, Germany; 2 Davee Department of Neurology and Clinical Neurosciences and Department of Physiology, Feinberg School of Medicine, Northwestern University, 60611Chicago, USA; 3 Department of Neurology and Institute of Translational Neurology, Westfälische Wilhelms-Universität, 48149 Münster, Germany; 4 Institute of Complex Systems, Zelluläre Biophysik (ICS-4), Forschungszentrum Jülich, 52425 Jülich, Germany; 5 Donders Centre for Cognition, Radboud University, 6500 Nijmegen, The Netherlands

**Keywords:** Delta oscillations, Thalamocortical oscillations, TRIP8b, HCN channels, Knockout mice, In vivo, *I*_A_

## Abstract

Hyperpolarization-activated cyclic nucleotide-gated cation (HCN) channels have important functions in controlling neuronal excitability and generating rhythmic oscillatory activity. The role of tetratricopeptide repeat-containing Rab8b-interacting protein (TRIP8b) in regulation of hyperpolarization-activated inward current, *I*_h_, in the thalamocortical system and its functional relevance for the physiological thalamocortical oscillations were investigated. A significant decrease in *I*_h_ current density, in both thalamocortical relay (TC) and cortical pyramidal neurons was found in TRIP8b-deficient mice (TRIP8b^−/−^). In addition basal cAMP levels in the brain were found to be decreased while the availability of the fast transient A-type K^+^ current, *I*_A_, in TC neurons was increased. These changes were associated with alterations in intrinsic properties and firing patterns of TC neurons, as well as intrathalamic and thalamocortical network oscillations, revealing a significant increase in slow oscillations in the delta frequency range (0.5–4 Hz) during episodes of active-wakefulness. In addition, absence of TRIP8b suppresses the normal desynchronization response of the EEG during the switch from slow-wave sleep to wakefulness. It is concluded that TRIP8b is necessary for the modulation of physiological thalamocortical oscillations due to its direct effect on HCN channel expression in thalamus and cortex and that mechanisms related to reduced cAMP signaling may contribute to the present findings.

**Electronic supplementary material:**

The online version of this article (10.1007/s00429-017-1559-z) contains supplementary material, which is available to authorized users.

## Introduction

The excitability of thalamic neurons is regulated by a number of peculiar ionic conductances generated by different voltage-gated ion channels such as low-threshold (T-type) Ca^2+^ channels (carrying *I*_T_), hyperpolarization-activated cyclic nucleotide-gated (HCN) channels (carrying the hyperpolarization-activated cation current, *I*_h_) and leak K^+^ channels which are the basis for the thalamic contribution in the genesis of EEG electroencephalogram delta waves during slow wave sleep (Sherman 2005; Timofeev and Bazhenov 2005; Bista et al. 2015a; Curró Dossi et al. 1992). Recurring interactions of these ionic conductances play an important role in the generation of single cell as well as network oscillations. In the absence of external synaptic inputs, for instance, TC neurons show a pattern of intrinsic rhythmic activity composed of bursts of action potentials (APs) at a frequency of 0.5–4 Hz (delta oscillations). The delta frequency rhythm in a single thalamocortical neuron is driven by the interplay of *I*_h_ and *I*_T_ (the latter is activated by *I*_h_-dependent depolarization). Synchronization of intrinsic membrane oscillations through synaptic mechanisms then leads to the network activities which characterize sleep and wake patterns of the EEG (McCormick and Bal 1997; Steriade et al. 1993; McCormick and Pape 1990).

Four HCN channel isoforms (HCN1-4) are the molecular basis of *I*_h_, which is inwardly directed at rest, and therefore, depolarizes the resting membrane potential (RMP). This current is involved in basic to more complex neuronal functions such as regulation of RMP, synaptic transmission, dendritic integration, initiation and control of rhythmic activity in neuronal circuits and contribution to subthreshold membrane potential oscillations (Wahl-Schott and Biel 2009; He et al. 2014). In fact, the role of *I*_h_ in the generation of single cell and synchronized network oscillations in the thalamocortical system during sleep and sensory processing has been analyzed in several studies (He et al. 2014; McCormick et. al 1992; McCormick and Pape 1990; Steriade et al. 1993; Wahl-Schott and Biel 2009; Llinas and Steriade 2006; Steriade and Deschenes 1984; Sherman and Guillery 2002; Kanyshkova et al. 2009; Curró Dossi et al. 1992). Dysregulation of *I*_h_ has been reported to play a role in some pathophysiological conditions including epileptic seizures and neuropathic pain (Herrmann et al. 2007; Kanyshkova et al. 2012; Wemhöner et al. 2015). The function of HCN channels is fine-tuned by several molecular factors. The modulatory role of these molecules is conveyed via direct effects on channels gating and/or through expression changes of the channel proteins. TRIP8b, also known as PEX5R and PEX5Rp, is a brain-specific auxiliary subunit for HCN channels which has been relatively recently discovered. Several different splice variants of TRIP8b have been uncovered which can differentially control the gating, surface expression and trafficking of HCN channel subunits in a subtype-specific manner (Santoro et al. 2004; Lewis et al. 2009; Santoro et al. 2009; Piskorowski et al. 2011; Huang et al. 2012). However, not much is known about the functional role of TRIP8b in physiological thalamocortical oscillations. A recent study (Heuermann et al. 2016) revealed that the complete loss of TRIP8b is associated with profound reduction of *I*_h_ and an absence epileptic phenotype thereby revealing pathophysiological thalamocortical activity. However, the effect of TRIP8b-deficiency on physiological oscillations in the thalamocortical system during natural sleep and wakefulness has not been addressed.

The aim of the present study was, therefore, to determine possible thalamus-related changes in brain rhythms, especially delta activity, by performing local field potential (LFP) recordings on freely moving TRIP8b^−/−^ and wild-type control mice in vivo. Since the occurrence of delta oscillations in the EEG critically depend on the maturation and properties of thalamic *I*_h_ (Kanyshkova et al. 2009), we analyzed this current in different thalamic nuclei. In addition, intrathalamic oscillations were investigated by performing LFP recordings in horizontal thalamic slices. Computer modeling approaches were used to assess the effects of graded changes in *I*_h_ on intrathalamic and thalamocortical oscillations. We found that the loss of TRIP8b induced a reduction of *I*_h_ in thalamic neurons which, in combination with reduced basal cAMP levels and increased *I*_A_, was associated with hyperpolarization of the membrane potential, increased bursting, slowed thalamocortical oscillations and increased delta activity during states of active-wakefulness thereby characterizing the TRIP8b-deficiency as thalamocortical dysrhythmia.

## Materials and methods

Mice with total elimination of TRIP8b in the brain, termed TRIP8b^−/−^ mice here, were obtained from the original colony at Davee Department of Neurology and Clinical Neurosciences and Department of Physiology (Northwestern University, Chicago, USA) and transferred to the Institute of Physiology I (Westfälische Wilhelms-Universität, Münster, Germany). Animals were three times backcrossed with C57BL/6J mice (Harlan Laboratories, Nienburg, Germany) and kept as knockout strain thereafter. As before (Lewis et al. 2011), C57BL/6J mice were used as controls (termed as wild-type, WT, in the following). All experimental procedures were performed in accordance with the principles approved by local authorities (review board institution: Landesamt für Natur, Umwelt und Verbraucherschutz Nordrhein-Westfalen; Approval IDs: 84-02.04.2015.A574, 84-02.05.50.15.026). Efforts were made to minimize the number of animals and the degree of discomfort to animals used in this study.

### Preparation of acute brain slices for patch-clamp recordings

Animals were sacrificed under isoflurane anesthesia and brain tissue was rapidly removed from the skull. Thalamic and cortical coronal slices (250 µm) were prepared from TRIP8b^−/−^ and WT mice of different ages (p15-30 and p90-120) in ice-cold oxygenated (O_2_) slicing solution, containing (in mM): sucrose, 200; PIPES, 20; KCl, 2.5; NaH_2_PO_4_, 1.25; MgSO_4_, 10; CaCl_2_, 0.5; dextrose, 10; pH 7.35, with NaOH. Slices were transferred to and kept in a chamber with artificial cerebrospinal fluid (ACSF) containing (in mM): NaCl, 120; KCl, 2.5; NaH_2_PO_4_, 1.25; NaHCO_3_, 22; MgSO_4_, 2; CaCl_2_, 2; glucose, 25. Temperature was set to 33 °C for 30 min and slices were allowed to cool down to room temperature thereafter. pH was adjusted to 7.35 by bubbling with carbogen (95% O_2_ and 5% CO_2_).

### Patch-clamp recordings in acute brain slices

*I*_h_ was characterized by whole-cell patch-clamp recordings from TC neurons of the ventrobasal complex (VB), posterior thalamic nucleus (PO), central-medial nucleus (CM) and the dorsal part of the lateral geniculate nucleus (dLGN), as well as pyramidal neurons in layer V and VI of the somatosensory cortex. Recordings were carried out in an external solution (bath solution) containing (in mM): NaCl, 125; KCl, 2.5; NaH_2_PO_4_, 1.25; HEPES, 30; MgSO_4_, 2; CaCl_2_, 2; Glucose, 10; BaCl_2_, 0.5–1 (only added in voltage-clamp recordings); pH 7.35, at 30 ± 1 °C. Patch pipettes were pulled from borosilicate glass (GC150T-10; Clark Electromedical Instruments, Pangbourne, UK) and had a resistance of 3–4 MΩ. The internal solution (pipette solution) contained (in mM): K-gluconate, 88; K_3_-citrate, 20; NaCl, 10; HEPES, 10; MgCl_2_, 1; CaCl_2_, 0.5; BAPTA, 3; Mg-ATP, 3; Na_2_-GTP, 0.5. The internal solution was set to a pH of 7.25 with KOH and an osmolality of 295 mOsmol/kg. All recordings were performed on the soma of TC or cortical pyramidal neurons using an EPC 10 amplifier (HEKA Elektronik, Lamprecht, Germany). The access resistance was in a range of 5–25 MΩ and was monitored throughout the experiment. Cells with access resistance more than 25 MΩ were discarded from the experiment. Series resistance compensation of > 30% was routinely applied. Voltage-clamp experiments were controlled by the software PatchMaster (HEKA Elektronik) operating on an IBM-compatible personal computer. All measurements were corrected offline for a liquid junction potential of 10 mV.

For voltage clamp recordings of K^+^ outward currents the extracellular solution contained (mM): NaCl, 125; KCl, 2; HEPES, 10; Glucose, 10; MgCl_2_, 4. For isolation of *I*_A_, TTX (1 µM) and tetraethylammonium (TEA-Cl, 10 mM) were added to the recording solution. CaCl_2_ was replaced by an equimolar concentration of MgCl_2_ and residual-free extracellular Ca^2+^ was chelated by adding EGTA (1 mM). The intracellular solution contained: NaCl 10, KCl 10, K-gluconate, 88; K_3_-citrate, 20; NaCl, 10; HEPES, 10; MgCl_2_, 1; CaCl_2_, 0.5; BAPTA, 3; Mg-ATP, 3; Na_2_-GTP, 0.5. The internal solution was set to a pH of 7.25 with KOH and an osmolality of 295 mOsmol/kg. A liquid junction potential of 19 mV was taken into account here.

### Voltage-clamp recordings

The protocol used for assessment of *I*_h_ current was as described previously (Kanyshkova et al. 2012). Briefly, *I*_h_ current was measured by hyperpolarizing steps of − 10 mV increments from a holding potential of − 40 to − 130 mV. The fraction of open channels, *p*(*V*), yielding the steady-state activation curve of *I*_h_, was calculated by normalizing the mean tail current amplitudes in response to an additional step of 1000 ms to − 100 mV using the following equation:

$$p(V)\;\, = \;\,(I - I_{\hbox{min} } )/(I_{\hbox{max} } - \,I_{\hbox{min} } ),$$

where *I*_max_ and *I*_min_ represent the tail current amplitudes for the voltage step from − 130 mV to − 100 mV and − 40 mV to − 100 mV, respectively. *I*_h_ activation was fitted by Boltzmann equation of the following form:

$$p(V)\,\; = \,\;1/(1 + \text{exp}((V - V_{0.5} )/k)),$$

in which *V*_0.5_ represents the voltage of half-maximal activation and *k* the slope factor. The amplitude of *I*_h_ was calculated by subtracting the instantaneous current amplitude from the steady-state current. The density of *I*_h_ was calculated by dividing the *I*_h_ current amplitude at − 130 mV by the membrane capacitance obtained during whole-cell recordings. The time course of *I*_h_ activation in TC neurons was best approximated by a dual exponential equation as follows:

$$I_{\text{h}} (t)\;\, = \,\;A_{1} \text{e}^{( - t/\tau 1)} \, + \,A_{2} \text{e}^{( - t/\tau 2)}$$

where *I*_h_ (*t*) is the total amplitude of the current at time *t*, and *A*_1_ and *A*_2_ are the respective amplitudes of the components with fast (*τ*1) and slow (*τ*2) time constants.

Membrane outward currents in TC neurons were elicited by holding cells at a potential of − 69 mV followed by hyperpolarization to a conditioning potential of − 129 mV (2 s duration) before stepping to various test potentials (− 99 to + 21 mV; 10 mV increment; 200 ms duration). To allow isolation of *I*_A_, we took advantage of its fast inactivating nature and inserted a pre-pulse to − 39 mV (100 ms duration) between the hyperpolarizing condition pulse and the test pulse. Pre-pulse-sensitive currents were analyzed. Inactivation of *I*_A_ was investigated by holding neurons at − 69 mV and stepping to different conditioning potentials (− 129 to − 19 mV, 2 s duration, 10 mV increment), before stepping to a constant analyzing test potential of − 9 mV of 100 ms duration.

The conductance *G* was estimated from the peak outward current amplitude *I* (determined within the first 15 ms of the test pulse) of the pre-pulse-sensitive current (see Fig. 7a) as follows:

$$G\; = \;I/(V - E_{\text{k}} )$$

with *V* being the voltage of the test pulse and *E*_k_ representing the K^+^ equilibrium potential (− 109 mV under the current recording conditions). Activation curves were obtained by fitting a Boltzmann distribution of the following form to the normalized data points:

$$G/G_{\hbox{max} } \, = \,\;1/(1\, + \,\exp ((V - V_{h} )/k)),$$

with *G*_max_ being the maximal conductance, *V*_h_ the half-maximal activation and *k* the slope factor. Inactivation curves were obtained without pre-pulse subtraction and using *I*/*I*_max_ (with *I*_max_ being the maximal peak current amplitude).The time course of *I*_A_ inactivation in TC neurons was best approximated by a single exponential equation as follows:

$$I_{A} (t)\,\; = \,\;A\text{e}^{( - t/\tau )} ,$$

where *I*_A_ (*t*) is the amplitude of the current at time *t*, _A_ is the maximal current amplitude and *τ* the time constant.

### Current-clamp recordings

In this study, the firing pattern and membrane properties of TC neurons such as RMP, input resistance (*R*_in_), and the *I*_h_-induced voltage sag were characterized by a current-clamp recording protocol consisting of a series of hyperpolarizing and depolarizing current injections (− 200 to 500 pA) with 20 pA increments, from the resting membrane potential of the cells. The length of each pulse was 1 s. The resting membrane potential of TC neurons was measured during the step with zero current injection.

### Preparation of acute brain slices for field potential recordings

Twelve to 24 week-old male mice were anesthetized with isoflurane. Brains were rapidly removed from the skull and placed in ice-cold slicing solution containing (in mM): 234 sucrose, 11 glucose, 24 NaH_2_PO_4_, 10 MgSO_4_ and 0.5 CaCl_2_, equilibrated with carbogen. Horizontal slices (400 µm) were cut using a microtome (Leica VT 1200 s, Leica, Wetzlar, Germany) and incubated in an oxygenated incubation solution (32 °C) for at least 1 h prior to recording.

### Rhythmic burst activity recordings

Horizontal brain slices were transferred to an interface chamber and recordings were performed at 32 ± 1 °C. The superfusion solution consisted of (in mM): NaCl, 126; KCl, 2.5; NaHCO_3_, 26; NaH_2_PO_4_, 1.25; MgCl_2_, 1; CaCl_2_, 2; glucose, 10; pH 7.35. Rhythmic burst activity was induced through stimulation of the internal capsule (IC) using a pair of tungsten electrodes (with 50–100 MΩ resistance) and network activity was measured in the VB complex using a glass electrode (GC150T-10; Clark Electromedical Instruments, Pangbourne, UK) with a resistance of 0.5–2 MΩ. Burst firing was characterized by at least three high-frequency spikes with an intra-burst frequency of > 100 Hz and inter-burst interval of not more than 500 ms. Activity was analyzed in a time interval ranging from 50 to 100 ms up to 2–3 s after stimulation of the IC. In some experiments, ZD7288 was applied via the bath solution. In this case, recordings were performed by first measuring a stable baseline and then recording for 1 h after ZD7288 application with a stimulation frequency of 1 impulse per minute. All analyses were performed offline using Clampfit 9.2 and Peak v1.0 software.

### Electrode implantation and LFP recordings for in vivo electrophysiology

Three to 5-month old male TRIP8b^−/−^ and WT mice were used for the in vivo experiments. Animals were anesthetized with an i.p. injection of 50 mg/kg pentobarbital supplemented by a subcutaneous injection of Carprofen (Rimadyl; 5 mg/kg). The head was fixed in a stereotactic frame (David Kopf Instruments, USA) and holes were drilled into the skull on top of the right hemisphere for the insertion of isolated (except at the tip) stainless steel wire recording electrodes (with the diameter of 0.127 mm; Franco Corradi, Milan, Italy) at the following positions: somatosensory cortex: *A*/*P* = 0, *M*/*L* = 3, depth = − 1.2; and ventral posterior medial nucleus (VPM): *A*/*P* = − 1.7,* M*/*L* = − 1.5, depth = − 2.8. Two epidural silver wires placed on top of the cerebellum served as ground and reference electrodes, respectively. The electrode assembly was fixed to the skull with dental acrylic cement (Pulpdent-GlassLute, Corporation Watertown, MA; USA). Following surgery, mice were allowed to recover for at least 1 week. Differential LFP signals from cortex and thalamus were recorded continuously for 8 h starting at 8 am during the light phase of the 12–12 h light–dark cycle. Recordings were performed in Plexiglas registration boxes (30 × 20 × 20 cm^3^). Animals were connected to the recording set up via a cable connected to a swivel allowing the animals to move freely during recording. The LFP signals were amplified with a physiological amplifier (Science Products DPA-2F), filtered by a band-pass filter with cut-off points at 1 (high pass) and 100 (low pass) Hz and digitalized with a constant sample rate of 2 kHz by a CED recording-system (Cambridge Electronic Design, UK). The behavioral activity of the animals was registered in parallel with the LFP recordings by a Passive Infrared Registration system (PIR, RK2000DPC LuNAR PR Ceiling Mount, Rokonet RISCO Group S.A., Belgium) placed on top of the registration box (van Luijtelaar et al. 2012). Following LFP recordings, animals were deeply anesthetized with an overdose of pentobarbital (i.p. injection) and the brain was removed for histological examination of the exact electrode position.

### Offline analysis of LFP recordings

The offline analysis of the recordings was carried out using NeuroExplorer 4 (Nex Technologies, USA) and Spike2 (version7.08, Cambridge Electronic Design Limited, UK) software. Only epochs from the first 2 h of the light period were selected for analysis; these hours represent the periods with the largest amount of specifically deep non-REM sleep (Huber et al. 2000). LFP data from cortex and thalamus from 20 epochs of 10 s duration of active-wakefulness and deep non-REM sleep of each animal were selected based on the EEG and PIR activity according to commonly used criteria (van Luijtelaar et al. 2012). For non-REM sleep, criteria included high-amplitude cortical EEG together with slow (1–5 Hz) waves in a motionless animal (as established by the PIR signal). For active-wakefulness criteria included behavioral activity (high and variable PIR signal accompanied by low-amplitude cortical EEG with theta and or beta) for the WT mice; in TRIP8b^−/−^ the PIR signal was only used since a dissociation between EEG and behavior was noticed (see results and discussion) and a high-amplitude and changing PIR signal was the only criterion used to establish active-wakefulness. The power spectral density (PSD) of all these epochs of non-REM sleep and active-wakefulness were calculated and averaged for each individual animal, the power density of the peak frequency of the conventional frequency bands (*δ* 1–4 Hz, *θ* 4.5–8 Hz, *α* 8.5–11, and *β* 11.5–30) was used for statistical analysis. To control for individual differences in EEG amplitude and PSD’s, relative values (*Z*-transformed) and percentages were analyzed.

### Histological verification of electrode positions

Correct placement of the electrodes was examined at the end of each experiment. Animals were deeply anaesthetized with isoflurane and a direct current of 9 V, 25 μA, with 10 s duration was passed through each electrode. Then animals were perfused with 4% phosphate-buffered paraformaldehyde (PFA). Brains were removed and post-fixed overnight in 4% PFA and later in 30% sucrose for 48–72 h. Free-floating coronal sections (40 µm) were cut with a microtome and stained with Cresyl-violet. Only animals with confirmed electrode positions were included for analysis.

### Drugs

Modulation of *I*_h_ by cyclic nucleotides was measured using different concentrations of 8-bromo adenosine-3′, 5′-cyclic monophosphate (8-Br-cAMP) sodium salt (Sigma-Aldrich, Germany), applied intracellularly through the recording pipette. The effects of 2-Chloro-*N* 6-cyclopentyladenosine (CCPA; Tocris Bioscience, UK), an adenosine A1 receptor agonist, was assessed by bath application of 100 µM of the compound. For experiments performed in the presence of SQ22536 (Sigma-Aldrich, Germany), an adenylyl cyclase inhibitor, slices were incubated for at least 1.5 h in 200 µM of the compound. In some experiments, *I*_h_ was abolished using different concentrations of ZD7288 (Tocris Bioscience, Bristol, United Kingdom) which was applied extracellularly. To reduce the excitability of cortical pyramidal neurons during voltage-clamp recordings, 0.5 µM of TTX (Merck, Darmstadt, Germany) was added to the external solution. For isolation of *I*_A_, we used 1 mM EGTA and 10 mM TEA-Cl (Sigma-Aldrich, Germany).

### Quantitative PCR

Real-time PCR was performed using the Taq-Man universal PCR master mix (Thermo Fisher Scientific, Massachusetts, USA) and the ABI Prism 7000 sequence detection system (Thermo Fisher Scientific, USA) (Budde et al. 2005; Kanyshkova et al. 2014). The PCR program was as follows: 2 min at 50 °C, 10 min at 95 °C, 50 cycles of 15 s at 95 °C and 1 min at 60 °C. Results were analyzed with ABI Prism 7000 SDS software. Quantification was done using the comparative *C*_T_ or ΔΔ*C*_T_ method as described in the ABI User Bulletin#2 (Thermo Fisher Scientific). Hybridization primer/probe assays for real-time PCR detection of *hcn*1, *hcn*2, *hcn*3, and *hcn*4 were purchased from Thermo Fisher Scientific.

### Western blotting

Brain tissues were homogenized in ice-cold lysis buffer containing 1% Triton, 150 mM of NaCl, 50 mM of TRIS base, pH 7.5, with protease inhibitors (Complete Ultra tablets, Mini, EDTA-Free, Easypack; Sigma-Aldrich, Germany) using a glass Teflon homogenizer followed by three times vortex mixing with 10 min intervals. The homogenate was centrifuged for 15 min, 14,000×*g* (4 °C), and the supernatant was used for Western blot analysis. Samples (10 µg protein) were loaded and separated by a 7.5% sodium dodecyl sulfate polyacrylamide gel (SDS-PAGE). Proteins were transferred onto nitrocellulose membrane at 20 V overnight. Membranes were incubated in a blocking buffer containing 5% non-fat milk in 1× TBS buffer (50 mM of Tris–HCl, 150 mM of NaCl) containing 0.1% (w/v) Tween 20 (TBST), pH 7.4 for 3 h at room temperature on a shaker and then incubated for 48 h at 4 °C with primary antibodies [polyclonal rabbit (rb)-anti-HCN1 (1:100); rb-anti-HCN2 (1:100); and rb-anti-HCN4, (1:100)]. Antibodies were purchased from Alomone Labs (Jerusalem, Israel). Membranes were washed three times for 10 min in washing buffer containing 1% (w/v) non-fat milk in TBS, and incubated with horseradish peroxidase-conjugated goat anti-rabbit secondary antibodies (polyclonal goat (gt) anti-rb HRP 1:1500; Dako, Denmark) in 1% (w/v) non-fat milk in TBST for 1 h at room temperature and washed (three times for 10 min) in TBST buffer thereafter. Bound antibodies were visualized using an enhanced chemiluminescent detection system. Quantification of HCN channel expression was by densitometric analysis of corresponding protein bands using ImageJ software. HCN channel signals were normalized to β-tubulin (anti-β-tubulin, 1:500, Abcam, UK) that was stained as an internal control and loading standard.

### Immunofluorescence

Twenty-five to thirty-day-old TRIP8b^−/−^ and C57BL/6J (WT) mice were transcardially perfused with 4% PFA. Brains were removed and post-fixed for 2 h in 4% PFA and later in 30% sucrose for 48–72 h. Free-floating coronal sections (40 µm) were cut and slices were collected in phosphate-buffered saline (PBS). Sections were washed three times for 10 min in PBS, preincubated for 20 min in PBS containing 0.3% (w/v) Triton-X100 (PBST). Slices were then incubated for 2 h in 6% (v/v) normal goat serum in PBST and incubated with primary antibodies: polyclonal rb-anti-HCN1, rb-anti-HCN2, rb-anti-HCN4 (1:200; Alomone Labs, Israel) and mouse (ms)-anti-TRIP8b (1:50; NeuroMab, USA) for 48 h at 4 °C. After incubation with the primary antibody, slices were washed three times for 10 min in PBS and thereafter transferred to the secondary antibody solution (Alexa Fluor 568 gt-anti-rb-IgG, 1:1000 and Alexa Fluor 488 gt-anti-ms-IgG, 1:1000) for 1 hour, washed three times for 10 min and mounted with a mounting medium (VECTASHIELD with DAPI, Vector Laboratories Inc., Burlingame, CA, USA) for confocal microscopy.

### Quantification of cAMP levels in tissue samples

Tissue from WT and TRIP8b^−/−^ mice was dissected from sacrificed animals in ice-cold buffer containing (in mM): sucrose, 200; PIPES, 20; KCl, 2.5; NaH_2_PO_4_, 1.25; MgSO_4_, 10; CaCl_2_, 0.5; dextrose, 10; pH 7.35 (with NaOH) and further incubated in an oxygenated salt solution (in mM: NaCl, 125; KCl, 2.5; NaH_2_PO_4_, 1.25; HEPES, 30; glucose, 10; CaCl_2_, 2; MgCl_2_, 1; IBMX, 0.05,pH 7.3) for 2 h. Samples were snap frozen in liquid nitrogen and stored at −80 °C before further use. All samples were thawed on ice and vortexed for 1 min at room temperature. To get rid of any non-solubilized material, samples were centrifuged at 1000×*g* for 10 min at 4 °C. The supernatant was transferred to a new Eppendorf cup and kept on ice. The amount of cAMP was determined with the cAMP-Screen assay kit (Applied Biosystems/ThermoFisher Scientific, Dreieich, Germany) following the supplier’s protocol. Determinations were performed in triplicates using 60 µl of tissue homogenate per assay. The protein content of each sample was determined by the Bradford Protein Assay (Bio-Rad, Munich, Germany). Values of cAMP were given per mg total protein. Calculations were done with GraphPadPrism 5.04 software (GraphPad, San Diego, CA, USA).

### Mathematical modeling

Simulations were conducted within the NEURON simulation environment (Hines and Carnevale 2001; Meuth et al. 2005) based on a modified version of an intrathalamic and a thalamocortical network model consisting of four and eight cells, respectively (Destexhe et al. 1996, 1998). The four-cell model comprised two spontaneously pacemaking TC neurons and two reticular thalamic (nRT) neurons interacting via GABA_A_, GABA_A+B_ and AMPA synapses (see Fig. 9a). While nRT neuron parameters were not changed, the *I*_h_ module of both TC neurons was modified by introducing activation kinetics and conductance obtained from our voltage-clamp recordings in nine WT and ten TRIP8b^-/-^ cells. Four different cell types were used for the eight-cell model (Destexhe et al. 1998), namely two cortical pyramidal neurons (PY), two cortical interneurons (IN), two TC neurons and two reticular thalamic neurons. The connectivity of the four different cell types is shown in Fig. 9d. As in the four-cell model, *I*_h_ parameters obtained from voltage-clamp recordings in TC and PY neurons were used in eight-cell simulation of network activity.

### Statistics

All results are presented as mean ± SEM. Comparison of *I*_h_ characteristics between TRIP8b^−/−^ and WT cells in different regions of the brain was performed by separate Student’s *t* tests. In case of multiple comparisons, such as in cAMP dose–response study or CCPA and SQ22536 studies either ANOVA or repeated-measures ANOVA were used. For in vivo experiments, comparison of the PSD between different states of vigilance, frequency bands and strains was done with Repeated-Measures ANOVA, in which the state of vigilance (wakefulness, deep non-REM sleep) and frequency band were set as within subject factors and groups (WT and TRIP8b^−/−^) as between-subject factor. For comparisons within the state of vigilance, groups and frequency band served as independent variables (between and within subjects factors, respectively) for the two-way repeated-measures ANOVA. Student’s *t* tests were used as Post Hoc tests, if necessary. Separate analyses were done for the cortical and thalamic PSD. Data analysis and figure plotting were performed by IBM SPSS Statistic software (version 22, USA), Corel DRAW X4 and OriginPro (version 8G, OriginLab, Friedrichsdorf, Germany) software.

## Results

### TRIP8b^−/−^ mice show slower cortical and thalamic oscillations during active-wakefulness

**Fig. 1:**
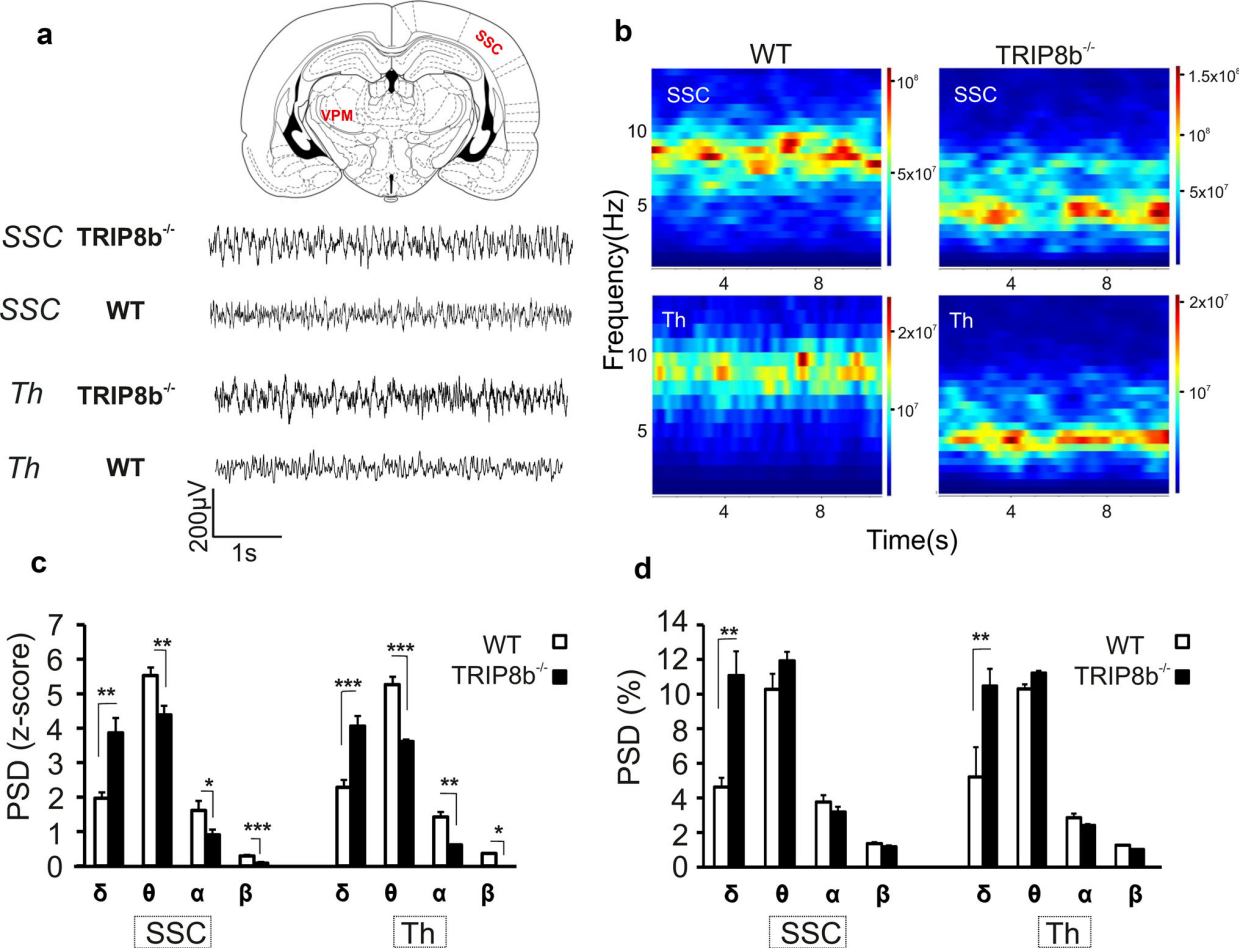
Modulation of thalamocortical oscillatory activities during active-wakefulness by TRIP8b. **a** Sample LFP recordings (5 s epochs) from somatosensory cortex (SSC) and ventral–posterior–medial nucleus (VPM) of the thalamus (Th) of TRIP8b^−/−^ and WT animals during active-wakefulness, indicating an increase in the power of delta oscillations in TRIP8b^−/−^ mice compared to WT animals. **b** Example of time–frequency analysis of cortical and thalamic oscillatory activities (each plot shows an average of 20 epochs of 10 s duration) for a single animal during active-wakefulness for WT and TRIP8b^−/−^ mice. As illustrated in these time-frequency plots, the LFP of WT during active-wakefulness in SSC and Th mainly contains frequencies in the range of 6–11 Hz with a peak power around 7-10 Hz. However, in TRIP8b^−/−^, the dominant frequencies during wakefulness range between 2 and 7 Hz in SSC and Th with a peak power at 3–4 Hz. **c** Bar graphs comparing the normalized peak PSD (z-score) of four frequency bands (δ–β) between WT (white bars) and TRIP8b^−/−^ (black bars) mice (n = 7/7). **d** Bar graphs comparing the percentage of PSD (PSD %) of four frequency bands (δ–β) between WT (white bars) and TRIP8b^−/−^ (black bars) mice. Mixed repeated-measures ANOVA followed by Student’s *t* tests. *,**, *** indicate *p* < 0.05, *p* < 0.01, *p* < 0.001, respectively

**Fig. 2:**
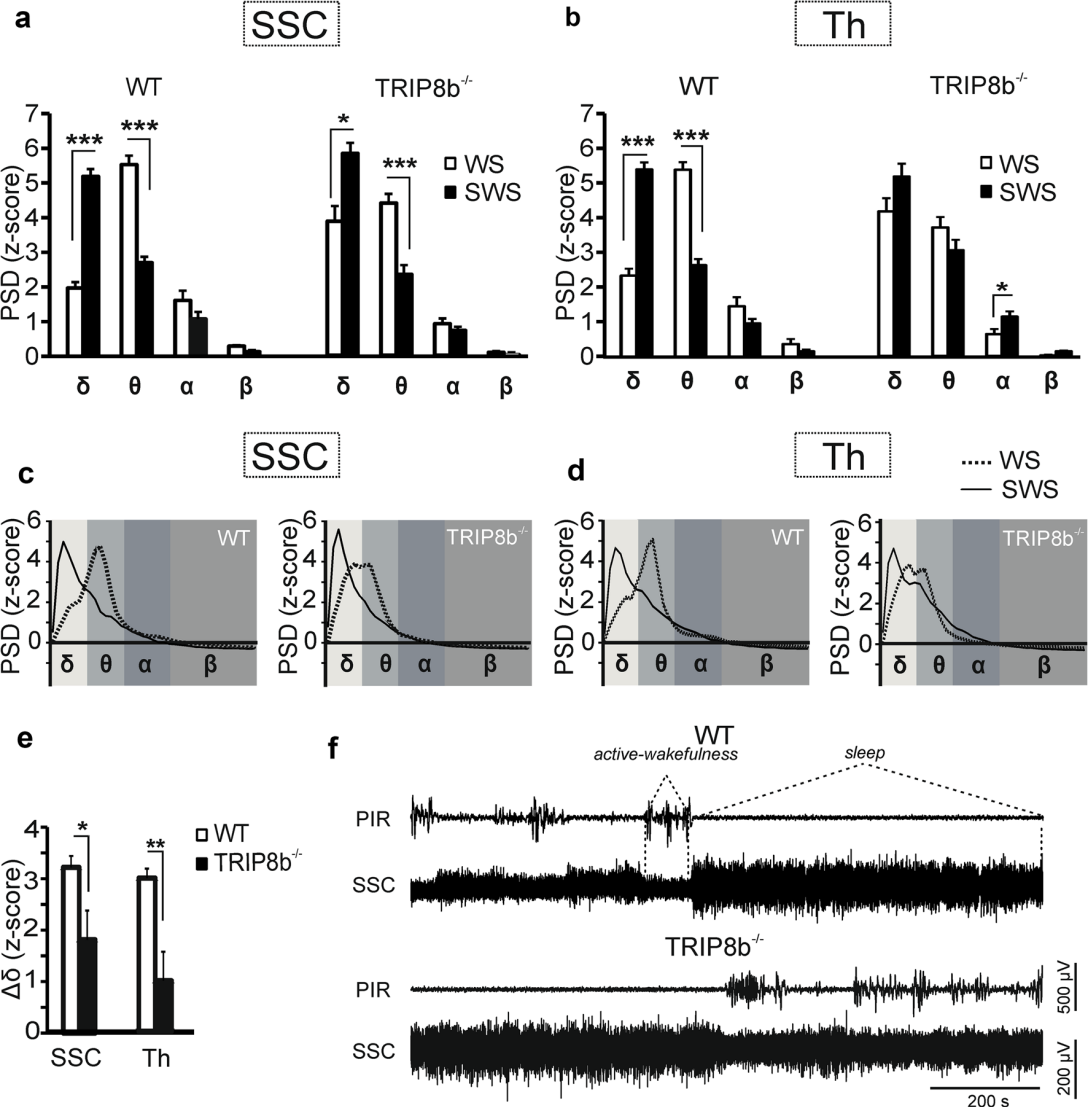
Modulation of cortical and thalamic slow-frequency oscillations by TRIP8b during episodes of slow-wave sleep and active-wakefulness. **a** and **b** Bar graphs comparing the normalized peak frequency PSD (z-score) of four frequency bands (δ–β) in somatosensory cortex (SSC) and VPM (Th) of WT and TRIP8b^−/−^ mice between episodes of active-wakefulness (WS) and slow-wave sleep (SWS). (mixed repeated measures ANOVAs followed by Student’s *t* tests, *n* = 7/7, *, **, *** indicate *p* < 0.05, *p* < 0.01, *p* < 0.001, respectively). **c** and **d** Representative spectrograms indicating the difference between the PSD during WS and SWS. **e** Bar graph comparing the changes in delta frequency oscillations (Δ*δ*) between the deep non-REM sleep (SWS) and active-wakefulness in WT and TRIP8b^−/−^ mice. As illustrated, TRIP8b^−/−^ mice (*n* = 7) show less (Student’s *t* tests, *p* < 0.05 for SSC and *p* < 0.01 for thalamus) changes in Δ*δ* compared to WT (*n* = 7) mice. **f** Sample LFP recordings from the SSC of WT (upper panel) and TRIP8b^−/−^ (lower panel) mice, showing the EEG signal during non-REM sleep and active-wakefulness in combination with the signal recorded from infrared movement detector (PIR). Note the smaller difference in the amplitude of the LFP between deep non-REM sleep and active-wakefulness in TRIP8b^−/−^ compared to WT mice. Dashed lines indicate episodes of sleep and active-wakefulness detected with the aid of both PIR and LFP signal

The two firing patterns of thalamocortical neurons, namely tonic and burst firing, are generated as a result of a complex interplay between different ionic conductances including *I*_h_, *I*_T_ and conventional *I*_Na_. Especially, bursting is involved in the generation of thalamocortical rhythms such as delta and spindle oscillations which are found in EEG recordings during non-rapid eye movement (non-REM) sleep and anesthesia (Steriade et al. 1993; Timofeev and Bazhenov 2005). Therefore, dysregulation in any of these ionic conductances is expected to induce changes in thalamocortical oscillations. Given the importance of TRIP8b in regulation of *I*_h_ in both TC and cortical pyramidal neurons (Heuermann et al. 2016), we assessed the effects of TRIP8b deletion on thalamocortical network oscillations by performing LFP recordings from thalamus and cortex of freely moving mice (Fig. 1a). The LFP during non-REM sleep seemed normal in TRIP8b^−/−^ mice (see Fig. 2f). In contrast, the signal during active-wakefulness in TRIP8b^−/−^ mice, as was unambiguously determined with the aid of the passive infrared registration (PIR) signal (van Luijtelaar et al. 2012), differed from that in WT (see Figs. 1a and 2f) with the cortical and thalamic EEG mimicking non-REM sleep. Comparison of the power spectral density (PSD) of the different frequency bands between TRIP8b^−/−^ and WT mice during episodes of active-wakefulness demonstrated more (ANOVA’s followed by Student’s *t* tests, *p*’s < 0.05, *n* = 7/7) delta power in cortical and thalamic LFPs and less (*p*’s < 0.05, *n* = 7/7, see Fig. 1c) energy in the cortical and thalamic LFP’s of theta, alpha and beta bands in TRIP8b^−/−^ compared to WT mice. The percentage of delta power (Fig. 1d, *p*’s < 0.05 for both cortex and thalamus) was also higher in TRIP8b^−/−^ than in WT mice. In contrast, no significant differences between the two groups were observed in the power values of LFPs during non-REM sleep (Fig. 2a, b).

It is known that during non-REM sleep delta oscillations are the predominant oscillatory activities of the brain with a higher PSD than during wakefulness, and that during active-wakefulness these slow oscillations are replaced by faster oscillations (theta, beta and gamma) with peak amplitudes in the theta frequency range. Therefore, in an intact thalamocortical system, the EEG desynchronizes during (active) wakefulness. This implies that a predominantly high-amplitude low-frequency EEG during non-REM sleep is replaced by low-amplitude high frequencies characterizing wakefulness. Although both TRIP8b^−/−^ and WT mice had a higher delta power density during non-REM sleep compared to active-wakefulness (Fig. 2a and b), TRIP8b^−/−^ mice showed significantly less-pronounced changes in thalamic and cortical delta frequency oscillations compared to WT mice, as was revealed by a smaller difference in scores between deep non-REM sleep and active-wakefulness (Fig. 2c–f) in TRIP8b^−/−^ mice. Therefore, the deletion of TRIP8b suppressed the typical desynchronization response of the brain which normally happens at the transition from non-REM sleep to wakefulness.

The occurrence of absence epileptic activity in TRIP8b-deficient mice has been reported before (Heuermann et al. 2016). Here, we did not find clear patterns of trains of high-amplitude sharp spikes and slow waves characterizing absence seizures (spike-and-wave discharges, SWDs) in established rodent absence epilepsy models (van Luijtelaar and Sitnikova 2006; Arain et al. 2015). However, some isolated short (< 1 s) irregular spindle complexes were found in 2 out of 9 animals with the incidence of total of 2 to 3 spike-like activities in 8 h recording period.

In summary, the EEG analyses revealed that delta oscillations are prominent in waking TRIP8b-deficient mice and that the desynchronization response is more subtle than in WT mice. This identifies TRIP8b as a molecule which contributes to acceleration and desynchronization of thalamocortical oscillations.

### TRIP8b does not change *hcn* channel gene expression pattern but regulates HCN protein expression in the thalamocortical system

**Fig. 3:**
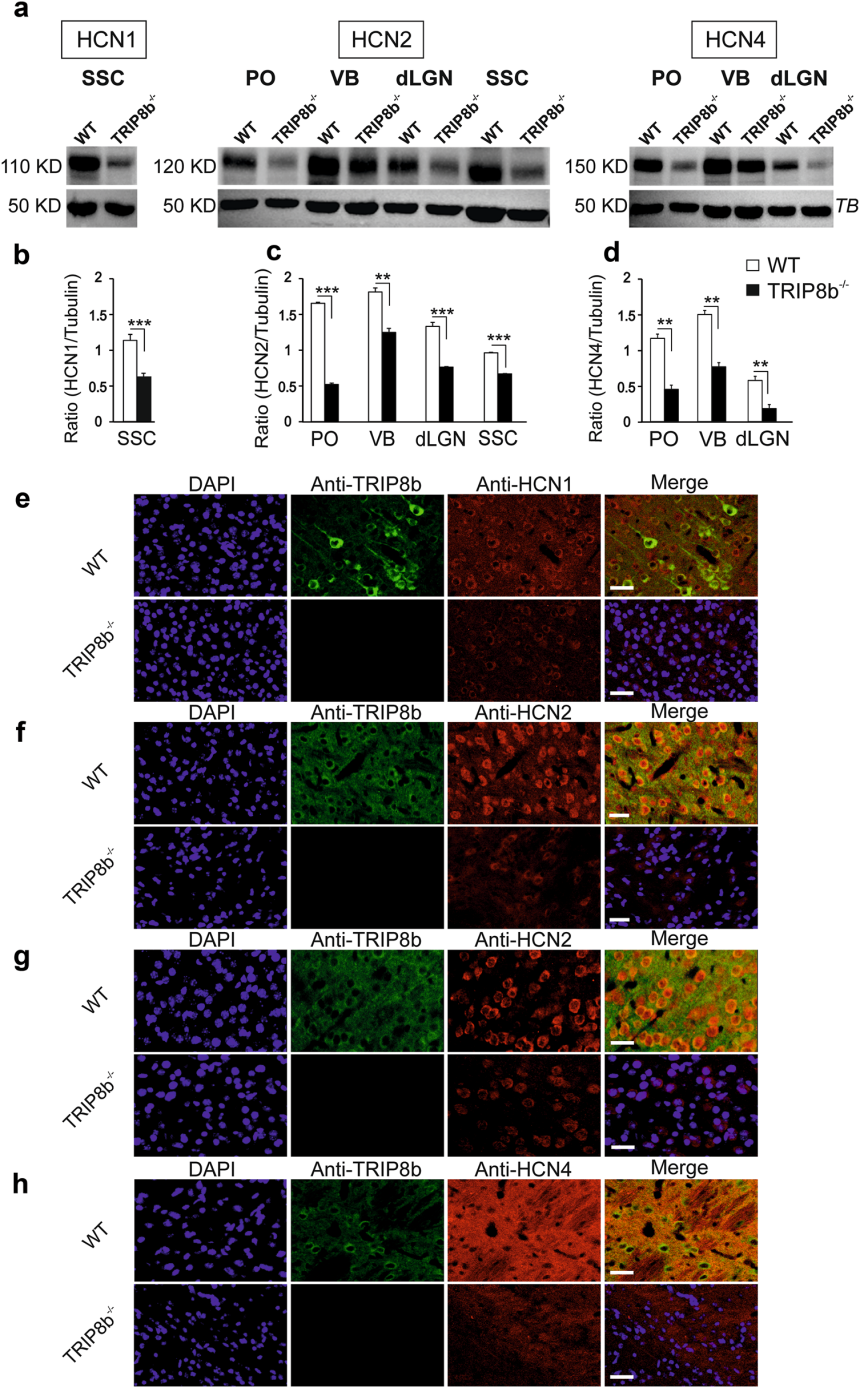
Modulation of HCN channel expression in thalamus and somatosensory cortex by TRIP8b. **a** For Western blotting, a total amount of 10 µg of protein was separated by SDS-PAGE and transferred onto nitrocellulose membrane. Blots were incubated with specific antibodies directed against HCN1 (rb-α-HCN1; 1: 100), HCN2 (rb-α-HCN2; 1: 100), HCN4 (rb-α-HCN4; 1: 100), and β-tubulin (anti-β-tubulin; 1: 500; TB). Bound primary antibodies were detected by gt-anti-rb HRP-coupled secondary antibodies and visualized by ECL. The size of the labeled bands is given in kilo Daltons (KD). In comparison to WT samples, the amount of HCN channel subunits (HCN1, 2 and 4) in different regions of thalamus and cortex of TRIP8b^−/−^ mice was reduced. SSC, PO, VB and dLGN represent somatosensory cortex, posterior thalamic nucleus, ventral–basal complex and dorsal part of the lateral geniculate nucleus, respectively. **b**, **c** and **d** Representative graphs displaying the ratio between HCN1, HCN2, HCN4 and β-tubulin (from left to right) between WT and TRIP8b^−/−^ mice (Student’s *t* tests, *n* = 4/4, **, *** indicate *p* < 0.01 and *p* < 0.001). **e**, **f**, **g**, **h** Immunohistochemical staining of HCN channel subunits in VB complex of the thalamus and cortex of WT and TRIP8b^−/−^ mice. DAPI staining was performed to identify the nuclei and is depicted in blue (first column); staining of TRIP8b (ms-anti-TRIP8b, 1:50) is depicted in green (second column); staining of HCN channel isoforms is depicted in red (rb-anti-HCN1, –HCN2 and –HCN4, 1:200, third column), merged images are depicted in the fourth column. **e** Specific labeling of HCN1 and TRIP8b in pyramidal neurons of layer V of the somatosensory cortex in WT and TRIP8b^−/−^ mice. Scale bar indicates 25 µm. **f** Specific labeling of HCN2 and TRIP8b in VB complex of WT (upper panel) and TRIP8b^−/−^ (lower panel) mice. The scale bar indicates 20 µm. **g** Specific labeling of HCN2 and TRIP8b in somatosensory cortex of WT (upper panel) and TRIP8b^−/−^ mice. Scale bar indicates 25 µm. **h** Specific labeling of HCN4 and TRIP8b in VB complex of WT and TRIP8b^−/−^ mice. Scale bar indicates 25 µm

Since TRIP8b is known to regulate the surface expression of HCN channels in different brain regions (Santoro et al. 2004, 2009), we assessed whether changes in thalamocortical oscillations are associated with altered *hcn* channel gene transcription and translation. We first quantified the mRNA levels of *hcn* 1–4 by qPCR in samples from cortex and thalamus dissected from WT and TRIP8b^−/−^ mice. No difference in gene expression was detected for any of the *hcn* genes in the somatosensory cortex (SSC), the posterior thalamic nucleus (PO), the ventral–basal complex (VB), the dorsal part of the lateral geniculate nucleus (dLGN) and the centromedial thalamic nucleus (CM) (Supplemental Figure 1a). In contrast, Western blot analysis demonstrated a significant reduction in the protein level of three HCN channel subunits (HCN1, HCN2, HCN4) in both the thalamus and cortex of TRIP8b^−/−^ mice compared to WT animals (Student’s *t* tests, *n* = 4/4 mice, *p*’s < 0.05, see Fig. 3a–d). The reduction in the protein level of different HCN channel subunits was accompanied by a reduction in the surface expression of the proteins, as illustrated by immunostaining with specific antibodies directed against HCN1, HCN2, HCN4 and TRIP8b on tissue sections encompassing regions of the thalamus, here PO, VB, dLGN, CM and cortex of TRIP8b^−/−^ and WT mice. As shown in Fig. 3e–h for WT animals, TRIP8b was detected in somata and dendrites of neurons in both the thalamus and layer V of the SSC, with overlapping expression profiles for HCN1 (in cortex), HCN2 (in thalamus and cortex) and HCN4 (in thalamus). Less dendritic expression of TRIP8b was detected in pyramidal neurons of layer VI (see Supplemental Figure 1b). Here, TRIP8b was mainly expressed in somata and strongly overlapped with HCN1 and HCN2 subunit expression. In TRIP8b^−/−^, mice a pronounced reduction in the general expression of HCN subunits in both cortex and different thalamic regions was observed (Fig. 3e–h and Supplemental Figs 1–4). In line with the results of previous studies (Heuermann et al. 2016), our immunohistochemistry data revealed that GABAergic neurons of the nRT did not express TRIP8b (Supplemental Figure 5a), which argues against significant changes of *I*_h_ in these neurons in TRIP8b^−/−^. In addition, immunostaining of the cortical and thalamic local circuit interneurons of GAD67/GFP knock-in mice with antibody against TRIP8b did not show any co-expression with TRIP8b in these neurons (see Supplemental Figure 5b–c). In these knock-in mice, the GABAergic neurons are labeled by expression of a green fluorescent protein (GFP) under control of the glutamate decarboxylase (GAD67/Gad1) promoter.

These results indicate that the regulatory effect of TRIP8b on HCN channels is not at the transcriptional level but rather via its role in controlling surface expression of the channels.

### Properties of *I*_h_ in cortical pyramidal neurons in the absence of TRIP8b

We used electrophysiological experiments to assess how reduced HCN channel expression influences *I*_h_ properties. Recordings of *I*_h_ currents were performed in pyramidal neurons of layers V and VI of the SSC. Measurements were carried out under voltage-clamp conditions. As shown in Supplemental Figure 5a and b, lack of TRIP8b resulted in a significant reduction in *I*_h_ current density in both cortical layers (layer V WT, 2.7 ± 0.4 pA/pF vs. layer V TRIP8b^−/−^ 1.2 ± 0.2 pA/pF, *n* = 8/9 cells, *p* < 0.001 and layer VI WT, 1.4 ± 0.3 pA/pF vs. layer VI TRIP8b^−/−^, 0.5 ± 0.1 pA/pF, *n* = 6/6 cells, *p* < 0.05, see Supplemental Figure 6b). In addition, analyses of steady-state activation curves in layer V pyramidal cells showed a significant negative shift in the voltage-dependent activation of *I*_h_ in TRIP8b^−/−^ mice compared to WT animals (layer V WT, *V*_0.5_ = − 90.6 ± 0.9 mV vs. layer V TRIP8b^−/−^, *V*_0.5_ = − 94.0 ± 1.0 mV, *n* = 8/9 cells, *p* < 0.05, Supplemental Figure 6c). Due to the rather small amplitude of *I*_h_ in layer VI neurons, a reliable calculation of *V*_0.5_ was not possible for these neurons. Therefore, I/V curves were generated by plotting mean *I*_h_ current density against the different hyperpolarizing step potentials (Supplemental Figure 6d). Data revealed a significant reduction in *I*_h_ current density in layer VI neurons from TRIP8b^−/−^ compared to WT animals (Mixed Repeated Measure ANOVA, *p* < 0.05, *n* = 6/6). When current kinetics were analyzed, no significant differences in the fast component of *I*_h_ were found between TRIP8b^−/−^ and WT neurons, suggesting that the time-dependent activation of HCN channels remained unaltered in TRIP8b^−/−^ mice. Only three out of nine recorded neurons from TRIP8b^−/−^ mice showed two-exponential activation time profiles. Therefore, a reliable comparison for *T*_2_ between the two groups was not possible. Reduction of *I*_h_ current density in layer V neurons also resulted in a significant negative shift in RMP (WT, RMP = − 68.4 ± 2.7 mV, *n* = 8 cells and TRIP8b^−/−^, RMP = − 76.9 ± 1.5 mV, *n* = 9 cells, *p* < 0.05) with no effects on RMP in layer VI (data not shown).

These data indicate that loss of TRIP8b reduces *I*_h_ current density and influences HCN channel steady-state activation properties in cortical neurons.

### Properties of *I*_h_ in different regions of the thalamus in the absence of TRIP8b

To assess the role of TRIP8b in the regulation of HCN channels in functionally different regions of the thalamus, *I*_h_ currents were examined in various nuclei of the thalamus, including PO, dLGN, CM, and VB complex. In the absence of TRIP8b, all TC neurons showed strongly reduced *I*_h_ amplitude and significant reduction in current density calculated at the hyperpolarization step to − 130 mV (WT VB, 11.75 ± 0.7 pA/pF, *n* = 9 cells, and TRIP8b^−/−^ VB, 2.3 ± 0.2 pA/pF, *n* = 10 cells, *p* < 0.001; WT PO, 8.5 ± 1.2 pA/pF, *n* = 9 cells, and TRIP8b^−/−^ PO, 2.7 ± 0.2 pA/pF, *n* = 8 cells, *p* < 0.01; WT CM, 4.5 ± 0.7 pA/pF, *n* = 8 cells, and TRIP8b^−/−^ CM, 1.5 ± 0.193 pA/pF, *n* = 6 cells, *p* < 0.01; WT dLGN, 7.1 ± 1.3, *n* = 9 cells and TRIP8b^−/−^ dLGN, 2.3 ± 0.2 pA/pF, *n* = 8 cells, *p* < 0.001; Supplemental Figure 7a and b).

Furthermore, the absence of TRIP8b was accompanied by a shift of the half-maximal activation of *I*_h_ (*V*_0.5_) to more hyperpolarized values (WT VB, − 84.7 ± 1.2 mV, *n* = 9 cells and TRIP8b^−/−^ VB, − 93.4 ± 1.2 mV, *n* = 10 cells, *p* < 0.001; WT PO, − 84.6 ± 1.1 mV, *n* = 9 cells and TRIP8b^−/−^ PO, − 90.3 ± 1.01 mV, *n* = 8 cells, *p* < 0.001; WT CM, − 83.2 ± 2.3 mV, *n* = 8 cells and TRIP8b^−/−^ CM, − 94.3 ± 2.5 mV, *n* = 6 cells, *p* < 0.001; WT dLGN, − 85.7 ± 2.2 mV, *n* = 9 cells and TRIP8b^−/−^ dLGN, − 92.1 ± 2.1, *n* = 8 cells, *p* < 0.05; see Supplemental Fig. 7b and c).

Since time-dependent activation kinetics represent a sensitive measure for the subunit composition and cyclic nucleotide-dependent modulation of HCN channels, we determined time constants of *I*_h_ activation. Under the current recording conditions, channel opening was best approximated by a double exponential equation. The deletion of TRIP8b slowed down both the fast (*T*_1_) and the slow (*T*_2_) components of time-dependent *I*_h_ activation. Results are presented in Supplemental Table 1.

Since HCN channel properties in the rodent thalamus change during development (Kanyshkova et al. 2009, 2012), *I*_h_ was assessed in TC neurons of 3–4-month old animals. The results presented in Supplemental Figure 8 show an age-dependent negative shift in the voltage-dependent activation of *I*_h_ (WT VB TC neurons, *V*_0.5_ from − 84.7 ± 0.8 mV in 15–30-day-old mice to − 91.2 ± 1.2 mV in 90–120-day-old animals, *n* = 9/6 cells, *p* < 0.001; TRIP8b^−/−^ VB TC neurons, *V*_0.5_ from − 93.4 ± 0.9 mV in 15–30-day-old to − 99.2 ± 2 mV in 90–120-day-old animals, *n* = 10/7 cells, *p* < 0.05; see Supplemental Figure 8a–d) accompanied by a slight increase in the current density (data not shown), in both TRIP8b^−/−^ and WT animals. In addition, *I*_h_ showed a slower activation time constant in TRIP8b^−/−^ of both age groups (see Supplemental Figure 8e).

These data indicate that TRIP8b regulates the functional expression of HCN channels and their steady-state and kinetic in thalamic neurons throughout postnatal development.

### Lower intracellular cAMP levels in the absence of TRIP8b

**Fig. 4:**
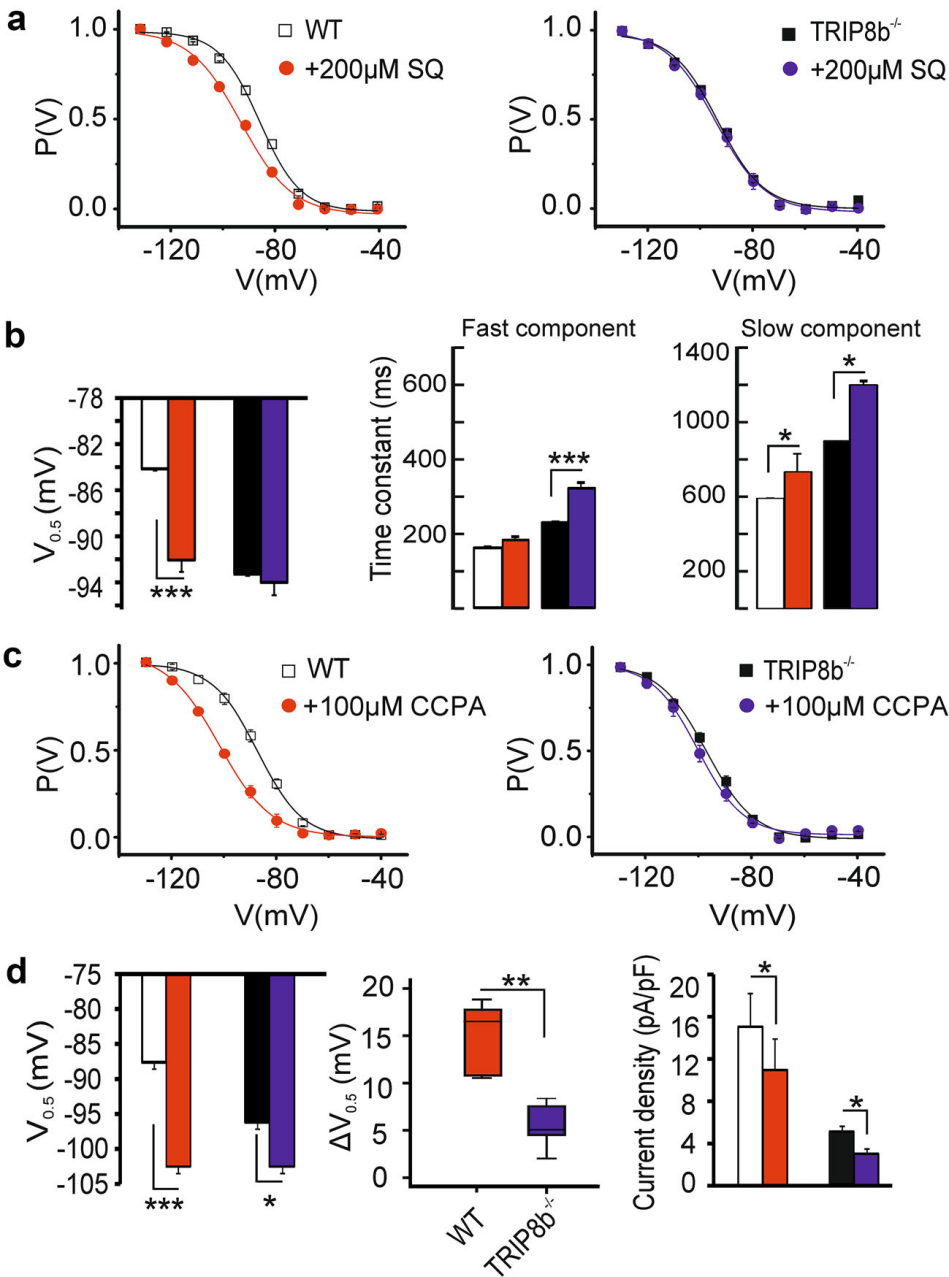
Modulation of I_h_ in TC neurons by cAMP. **a** Graphs showing the mean steady-state activation curves of *I*_h_ in thalamocortical (TC) neurons of WT (*n* = 8/7 cells) and TRIP8b^−/−^ (*n* = 11/8 cells) mice. Slices were incubated either with 200 µM (dots) or without (squares) the adenylyl cyclase inhibitor SQ22536. Incubation with SQ22536 shifted the voltage-dependent activation (*V*_0.5_) of *I*_h_ to more hyperpolarizing potentials without having any effects on *I*_h_ in TRIP8b^−/−^ TC neurons (left panel, ANOVA followed by Student’s *t* tests, *** indicates *p* < 0.001). **b** (middle and right panel) The activation kinetics of I_h_ in both TRIP8b^−/−^ and WT TC neurons were slowed down in SQ22536 treated cells. **c** Graphs showing the mean steady-state activation curves of *I*_h_ in WT (*n* = 5 cells) and TRIP8b^−/−^ (*n* = 6 cells) TC neurons of the VB complex following bath application of 100 µM CCPA (dots). **d** Although CCPA shifted the voltage-dependent activation of *I*_h_ to negative potentials in both groups (left panel), the hyperpolarizing shift in *V*_0.5_ of *I*_h_ (Δ*V*_0.5_, middle panel) in thalamic relay neurons of TRIP8b^−/−^ mice was significantly smaller compared to WT TC neurons. The reduction in *I*_h_ current density after application of CCPA in both groups is depicted in the bar graph (right panel). Repeated-measures ANOVA followed by Student’s *t* tests, *, **, *** indicate *p* < 0.05, *p* < 0.01, and *p* < 0.001, respectively

Since modulation by cAMP is a characteristic property of HCN channels and TRIP8b has been shown to interfere with this mechanism (Zolles et al. 2009; Hu et al. 2013), we manipulated intracellular cAMP levels in the following. Currents measured during application of different concentrations of 8-bromo (Br)-cAMP via the patch pipette. 8-Br-cAMP induced a dose-dependent depolarizing shift in voltage-dependent activation of *I*_h_ in both TRIP8b^−/−^- and WT-derived TC neurons (Supplemental Figure 9a–c). However, the effect of cAMP revealed differences with respect to sensitivity and efficacy in the two strains. Application of 8-Br-cAMP induced a pronounced positive shift in *V*_0.5_ already at lower concentrations in TRIP8b^−/−^ compared to WT mice, suggesting a higher cAMP sensitivity of HCN channels in the absence of TRIP8b. Interestingly, in both genotypes *V*_0.5_ at 100 µM 8-Br-cAMP was shifted to a similar depolarized value of − 66.3 mV in WT and − 66.1 mV in TRIP8b^−/−^ TC neurons (Supplemental Figure 9a and c; *n* = 9/6). The maximal depolarizing shift, however, was larger in TRIP8b^−/−^ neurons (Δ*V*_0.5_ = 27.2 mV; from − 93.3 to − 66.1 mV) than in WT neurons from Δ*V*_0.5_ = 18.4 mV; − 84.7 to − 66.3 mV indicating a higher cAMP efficacy in the absence of TRIP8b. In addition, 8-Br-cAMP induced an increase in *I*_h_ current density only in WT TC neurons without changing this parameter in TRIP8b^−/−^ TC neurons (Supplemental Figure 9d). Based on the increased cAMP sensitivity of *I*_h_ in TRIP8b^−/−^ mice, we expected their control values of *V*_0.5_ to be more depolarized under resting conditions in comparison to WT animals. To test for possible changes in the basal intracellular cAMP levels in TC neurons from TRIP8b^−/−^ mice, we used the adenylyl cyclase inhibitor SQ22536. Incubation of thalamic slices with SQ22536 (200 µM, 1.5 h) shifted the activation curve of *I*_h_ to more hyperpolarizing potentials in TC neurons of WT but not TRIP8b^−/−^ mice (ANOVA’s followed by Student’s *t* tests, WT: *V*_0.5_ from − 84.7 ± 0.8 to − 91.8 ± 1.2 mV, *n* = 8/7 cells, *p* < 0.001; TRIP8b^−/−^: *V*_0.5_ from − 93.0 ± 1.8 mV to − 93.7 ± 2.0 mV, *n* = 11/7 cells, *p* > 0.05; Fig. 4a). Furthermore, time constants of *I*_h_ activation were slower in SQ22536-treated groups as compared to non-treated controls (ANOVA’s followed by Student’s *t* tests, *p*’s < 0.05 for fast and slow components of *I*_h_ activation, see Fig. 4b).

Next, we tested the effect of the adenosine A_1_ receptor agonist CCPA which is known to cause adenylyl cyclase inhibition and thereby reducing intracellular cAMP levels (van Calker et al. 1979; Pape 1992). As shown in Fig. 4c, d, bath application of 100 µM CCPA shifted the voltage-dependent activation of *I*_h_ to more hyperpolarized potentials in both, WT and TRIP8b^−/−^ TC neurons. The effect was stronger for TC neurons from WT compared to TRIP8b^−/−^ mice (Fig. 4d, left panel; WT: *V*_0.5_ from − 87.6 ± 1.1 mV to − 102.5 ± 1.15 mV, *n* = 5 cells, *p* < 0.001; TRIP8b^−/−:^ *V*_0.5_ from − 96.18 ± 1.1 mV to − 102.5 ± 1.3 mV, *n* = 6 cells, ANOVA’s followed by Student’s *t* tests, *p*’s < 0.05). In addition, Δ*V*_0.5_, the shift in *V*_0.5_ produced by application of 100 µM CCPA, was significantly smaller in TC neurons from TRIP8b^−/−^ compared to WT mice (Fig. 4d, middle panel; Δ*V*_0.5_ = 6.3 ± 1.7 mV in TRIP8b^−/−^ and Δ*V*_0.5_ = 14.9 ± 1.76 mV in WT, *n* = 6/5 cells, p < 0.01). Moreover, application of CCPA decreased *I*_h_ current density measured at the step to − 130 mV (Fig. 4d, right panel; WT TC neurons: from 15.0 ± 3.1 pA/pF to 10.9 ± 2.9 pA/pF, *n* = 5 cells, *p* < 0.05, TRIP8b^−/−^ TC neurons: from 4.9 ± 0.7 pA/pF to 3.0 ± 0.5, *n* = 6 cells, *p* < 0.05) and also slowed down the fast and slow components of *I*_h_ activation kinetics in both groups (Repeated-measures ANOVA showed a significant main effect of CCPA application on time constants: *p* < 0.05 for fast and slow components of *I*_h_, respectively; data not shown).

In line with the results of a previous study (Heuermann et al. 2016), these results indicate an increased cAMP sensitivity of *I*_h_ in TC neurons from TRIP8b^−/−^ mice. Furthermore, there seems to be a lower basal adenylyl cyclase activity in TC neurons from TRIP8b^−/−^ mice which results in reduced basal cAMP concentrations, hyperpolarized RMP and smaller effects of G_i_-coupled receptors.

To further address this possibility, we assessed basal cAMP levels in brain tissue samples (Supplemental Figure 9e). Quantification of cAMP concentration from 3-month old WT and TRIP8b^−/−^ mice revealed a significantly lower cAMP level in knockout animals compared to WT animals (WT = 27.0 ± 4 pmol cAMP/mg protein, TRIP8b^−/−^ = 13.2 ± 2.1 pmol cAMP/mg protein, *n* = 3/3 animals, total of 18 samples, *p* < 0.01).

### Changes in firing pattern and intrinsic properties of TC neurons in TRIP8b^−/−^ mice

**Fig. 5:**
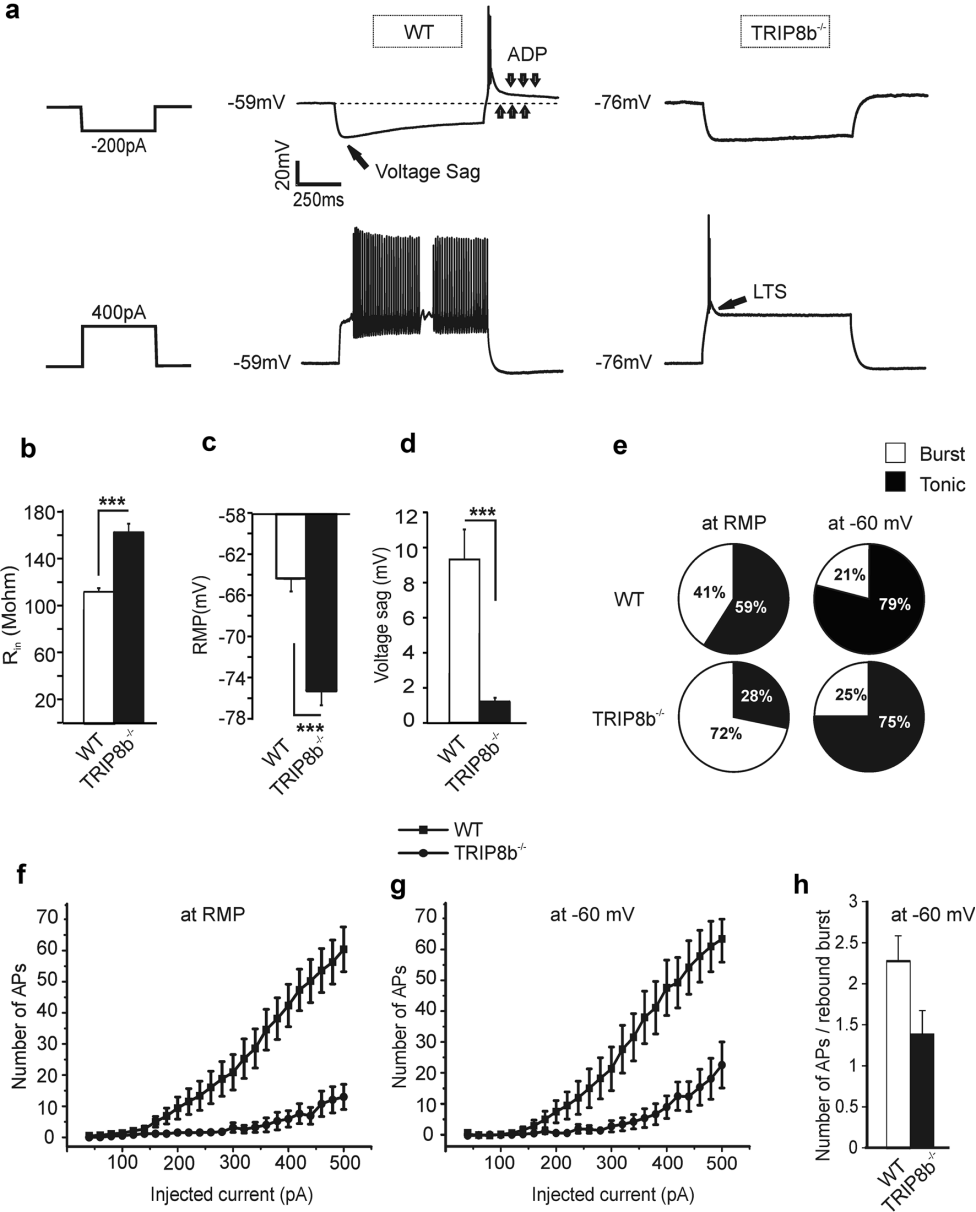
Changes in firing pattern and intrinsic properties of the TC neurons in TRIP8b^−/−^ mice. **a** Sample traces recorded in response to the injection of hyperpolarizing and depolarizing currents from the RMP of TC neurons in the VB complex of WT and TRIP8b^−/−^ mice. Note the different behavior of cells in response to the positive and negative current injections. LTS (see arrow, lower right panel) and ADP (3 downward arrows, upper panel) represent low-threshold Ca^2+^ spike and I_h_-dependent afterdepolarizing potential, respectively. The 3 upward arrows indicate RMP.  **b** and **c** show the higher R_in_ and significantly hyperpolarized RMP of TC neurons in TRIP8b^−/−^ compared to WT mice (Student’s *t* tests. ** and *** indicate, *p* < 0.01 and *p* < 0.001, respectively). **d** Bar graph showing a significant reduction in the *I*_h_-dependent voltage sag in TRIP8b^−/−^ (Student’s *t* tests, *** indicates *p* < 0.001).The voltage sag was measured upon injection of a hyperpolarizing current of -200 pA. **e** Pie charts indicating the percentage (%) of burst activity in TC neurons of TRIP8b^−/−^ (72%, *n* = 14 cells) compared to WT mice (41%, *n* = 22 cells), when cells were challenged with depolarizing currents from RMP (left panels). The occurrence of burst firing was no longer different between the two groups when TC neurons were held at a more depolarized potential of around − 60 mV by DC current injection (right panels). **f** The number of APs elicited by the injection of positive currents with 20 pA increments from RMP is shown. Significantly (Repeated-measures ANOVA, *p* < 0.001) less APs were evoked in TRIP8b^−/−^ TC neurons. **g** The number of depolarization-induced APs in TRIP8b^−/−^ TC neurons after compensation of the membrane hyperpolarization by injection of a small positive DC current (holding the RMP at − 60 mV) was still significantly (repeated-measures ANOVA, *p* < 0.001) lower compared to the WT TC neurons. **h** Bar graph comparing the number of APs on rebound burst in WT and TRIP8b^−/−^ VB TC neurons

To determine the functional impact of TRIP8b on passive membrane properties and neuronal firing, VB TC neurons were recorded under current-clamp conditions. Since tonic *I*_h_ activity influences passive membrane properties, we analyzed *R*_in_ and RMP. Indeed the reduction of *I*_h_ was accompanied by an increase in *R*_in_ (Fig. 5b; WT: 110.2 ± 3 MΩ; TRIP8b^−/−^: 160.5 ± 8 MΩ; *n* = 22/13 cells, *p* < 0.001) and a hyperpolarizing shift in RMP (Fig. 5c; WT: − 64.3 ± 1.9 mV; TRIP8b^−/−^: − 75.2 ± 1.8 mV, *n* = 22/13 cells, *p* < 0.001). The occurrence of the two prototypical firing patterns of TC neurons in rodents, namely burst and tonic firing, depends on the level of the prevailing membrane potential (Fig. 5a). Following depolarizing current pulses, TC neurons generate bursts of APs riding on top of a low-threshold Ca^2+^ spike (LTS; see arrow in Fig. 5a lower right panel) and tonic activity when the prevailing membrane potential is below − 70 mV and above − 60 mV, respectively (Cerina et al. 2015; Bista et al. 2015b; Llinas and Steriade 2006). In the voltage range between − 70 and − 60 mV burst and tonic firing may be intermingled. Bursting may also occur following release from a hyperpolarizing pulse in the form of a rebound burst (see Fig. 5a upper panel). During the course of negative voltage deflections, another hallmark of *I*_h_ activation is triggered, namely the depolarizing voltage sag (see arrow in Fig. 5a upper panel). In accordance with the reduction in *I*_h_, negative current injections (− 200 pA, 1000 ms) from the RMP induced voltage sags which were significantly smaller in the absence of TRIP8b (Fig. 5d; WT VB: 9.3 ± 2.2 mV; TRIP8b^−/−^ VB: 1.1 ± 1.7 mV; *n* = 22/13 cells, Student’s *t* test, *p* < 0.001). Interestingly, injection of hyperpolarizing currents to TC neurons of TRIP8b^−/−^ mice did not elicit the typical rebound burst and *I*_h_-dependent afterdepolarizing potential (ADP) which occurs due to deactivation of HCN channels (Fig. 5a upper right panel), probably because the threshold for activation of T-type Ca^2+^ channels is not reached due to the negative RMP. Indeed, rebound bursts reappeared with the same amount of hyperpolarizing current injections while holding the cells at − 60 mV, yet, the number of APs triggered by the LTS was lower in TRIP8b^−/−^ mice (Fig. 5h). When TC neurons in TRIP8b^−/−^ mice were challenged with depolarizing currents from RMP, burst activity was more frequently elicited in comparison to WT mice (Fig. 5a lower panel and e). As illustrated in Fig. 5e, injection of depolarizing currents to TRIP8b^−/−^ TC neurons from RMP resulted in burst activity in 72% of the cells (*n* = 14) compared to 41% in WT TC neurons (*n* = 22, *p* < 0.01). To further assess tonic firing properties, TC neurons were held at a more depolarized potential of around − 60 mV by DC current injection. Under these conditions, the occurrence of burst firing was no longer different between the two groups (Fig. 5e right panel). In accordance with the predominance of bursting, the total number of APs generated by depolarizing pulses was lower in TC neurons from TRIP8b^−/−^ in comparison to WT mice (Fig. 5f). However, TRIP8b^−/−^ TC neurons still showed a significantly lower total number of tonic APs than WT neurons after injection of depolarizing currents (Fig. 5g).

**Fig. 6:**
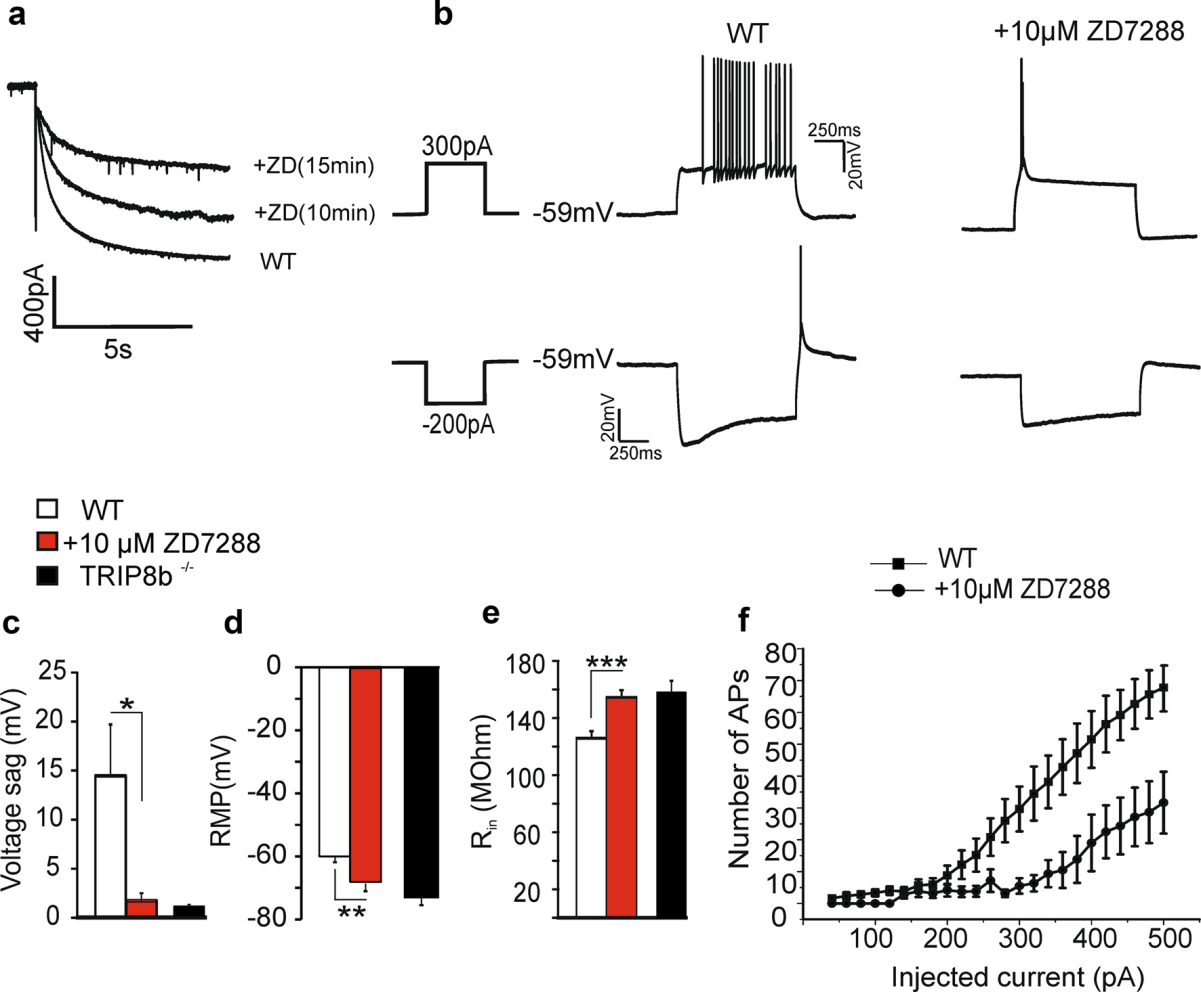
Reduction of *I*_h_ in thalamic relay neurons by ZD7288. **a** Sample traces recorded under voltage-clamp conditions in the presence of 10 µM ZD7288 from TC neurons of the VB complex of WT mice. The reduction of *I*_h_ after 15 min of blocker application resembles the *I*_h_ amplitude in TRIP8b^−/−^ mice (see Supplemental Figure 7). **b** Sample traces obtained from current-clamp recordings demonstrating the changes in firing pattern and RMP of TC neurons following application of 10 µM ZD7288. **c** Bar graph showing the significant reduction in the voltage sag upon current injection of − 200 pA (*n* = 6). The reduction in the voltage sag is comparable with the values obtained from TRIP8b^−/−^ relay neurons. The reduction in *I*_h_ density is accompanied by a significant (ANOVAs, *p* < 0.001) shift to hyperpolarizing potentials in RMP of WT TC neurons (**d**) and an increase in *R*_in_ (**e**). As shown in (**f**) the number of APs elicited by the injection of positive currents with 20 pA increment from RMP after bath application of ZD7288 (filled circles) is significantly smaller (ANOVAs, *p* < 0.001) than under control conditions (filled squares)

Since *I*_h_ density sets the level of the membrane potential, thereby influencing tonic firing patterns in thalamic interneurons (Leist et al. 2016), we tried to mimic the effects of TRIP8b loss by applying the HCN channel blocker ZD7288. To further strengthen the conclusion that the changes in RMP and firing properties seen in VB TC neurons in TRIP8b^−/−^ can be explained by reduction of *I*_h_, WT mice were investigated under current-clamp conditions in the presence of 10 µM ZD7288. This concentration was chosen based on previous experiments under voltage-clamp conditions where 10 µM ZD7288 reduced *I*_h_ current density to a level very similar to that found in TRIP8b^−/−^ mice (Fig. 6a). As illustrated in Fig. 6c–e, application of ZD7288 under current-clamp conditions resulted in a significant decrease in the *I*_h_ voltage sag (Fig. 6c; from 14.5 ± 5.21 mV to 1.7 ± 0.8 mV, *n* = 6 cells, *p* < 0.001) and a significant hyperpolarizing shift in the RMP (Fig. 6d; from − 60.03 ± 1.8 to − 69.8 ± 1.7 mV, *n* = 6 cells, *p* < 0.01), accompanied by an increase in the input resistance of the cells (Fig. 6e; from 125.8 ± 3.7 to 158.83 ± 4.23 MΩ, *n* = 6 cells, *p* < 0.001). Only two out of six cells showed a rebound burst after application of ZD7288 and the number of APs superimposed on the LTS was significantly lower (APs on LTS, 3.5 ± 0.8 before and 0.8 ± 0.5 after application of ZD7288, *n* = 6 cells, *p* < 0.05, data not shown). Moreover, ZD7288 increased the probability of burst firing from RMP after injection of positive currents (data not shown) and decreased tonic firing when cells were depolarized to − 60 mV (Fig. 6f; Repeated Measures ANOVA showed a significant interaction between groups and positive current injection, *p* < 0.001, and a significant main effect for groups *p* < 0.01). Previous studies have reported ZD7288 as a potent blocker of Nav1.4 (Wu et al. Wu et al. 2012) and in higher concentrations (> 100 µM) of low-voltage-activated calcium channels (Felix et al. 2003; Sánchez-Alonso et al. 2008), thereby characterizing this drug as a non-selective blocker for HCN channels. Considering that Nav1.4 channels are not present in rodent thalamic neurons (Kirchhof et al. 2015) and based on the fact that we applied 10 µM ZD7288 (thus having little effect on low-voltage-activated calcium channels), the reduction of tonic firing of TC neurons seems to be mainly based on the block of HCN channels.

These results indicate that TRIP8b is necessary for the generation of tonic currents through HCN channels, thereby influencing passive and active membrane properties of TC neurons. Furthermore, the effects of TRIP8b knockout on firing pattern and intrinsic properties of TC neurons are largely reproduced by pharmacological reduction of *I*_*h*_.

### Analysis of the fast transient potassium outward current in TRIP8b^−/−^ mice

**Fig. 7:**
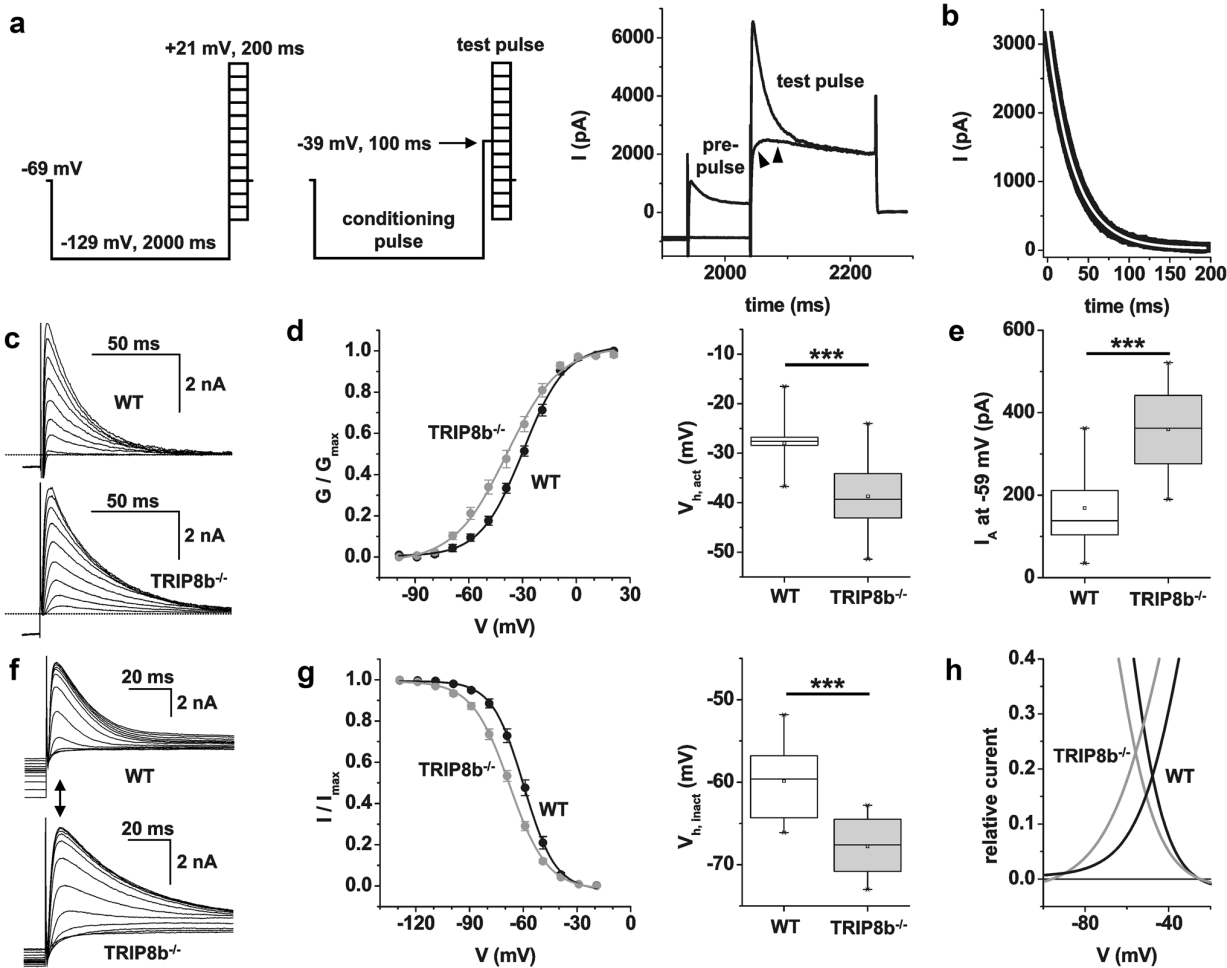
Voltage-dependent properties of *I*_A_ in TC neurons of WT and TRIP8b^−/−^ mice. **a** Voltage protocols to isolate *I*_A_. Cells were set to a conditioning potential (2 s duration) of − 129 mV before stepping to varying depolarizing potentials (− 99 to +21 mV, 200 ms duration; left panel) to evoke outward currents. Then, the voltage protocol was repeated with a pre-pulse to − 39 mV (100 ms duration; see arrow) inserted between the conditioning and the test pulse (middle panel). Inclusion of the pre-pulse resulted in complete disappearance of the transient outward current component during the test pulse (see arrow heads in the right panel; traces of test pulses to + 21 mV in a WT animal with and without pre-pulse are shown). The pre-pulse-sensitive current component was obtained by graphical subtraction of current with and without pre-pulse. **b** The decay of a pre-pulse-sensitive current to + 1 mV in a WT mouse is best fitted by a single exponential function with *τ* = 33 ms. **c** Families of transient outward currents obtained by graphical subtraction in WT (upper panel) and TRIP8b^−/−^ (lower panel) mice. Dashed lines indicate the zero line. **d** Activation curves of *I*_A_ in WT (black symbols) and TRIP8b^−/−^ (gray symbols) mice were obtained through Boltzmann fits of relative conductance values (left panel). Box plot representation of half-maximal activation values in WT (white box) and TRIP8b^−/−^ (gray box) mice (right panel). **e** Box plot representation of *I*_A_ current amplitudes at a test potential of − 59 mV in WT (white box) and TRIP8b^−/−^ (gray box) mice (right panel). **f** Inactivation of *I*_A_ was determined by varying a 2 s conditioning pulse between − 129 and − 19 mV, and keeping the following test potential constant at − 9 mV (200 ms duration). Families of transient outward currents in WT (upper panel) and TRIP8b^−/−^ (lower panel) mice are shown. Peak current amplitudes within 15 ms after stepping to the test pulse potential were determined (see double arrow). **g** Inactivation curves of *I*_A_ in WT (black symbols) and TRIP8b^−/−^ (gray symbols) mice were obtained through Boltzmann fits of relative current values (left panel). Box plot representation of half-maximal inactivation values in WT (white box) and TRIP8b^−/−^ (gray box) mice (right panel). **h** Combined Boltzmann fits (as in **d** and **g**) of activation and inactivation curves in WT (black lines) and TRIP8b^−/−^ (gray lines) mice reveal an area of overlap, the *I*_A_ window current. Please note that more current is available in TRIP8b^−/−^ mice

Since *I*_A_ is known to shape bursting as well as tonic firing of TC neurons (Pape et al. 1994; Kanyshkova et al. 2011), we characterized this outward current in the following. To elicit *I*_A_, cells were held at a potential of − 69 mV followed by hyperpolarization to a conditioning potential of − 129 mV (2 s duration) before stepping to various test potentials (− 99 to +21 mV; 10 mV increment; 200 ms duration; see Fig. 7a, left panel). To minimize the contribution of delayed rectifier K^+^ channels, we added TEA (10 mM) to the external solution. Furthermore, voltage-dependent Na^+^ channels were blocked with TTX (1 μM) and Ca^2+^ currents were eliminated using Ca^2+^-free (+ 1 mM EGTA) extracellular solutions (Budde et al. 1992). Under these conditions, outward current amplitudes rapidly increased with increasing step depolarization and the current waveform revealed a characteristic initial peak within less than 10 ms (Fig. 7a, right panel). To allow isolation of *I*_A_, a pre-pulse protocol was used (Fig. 7a, middle panel). Therefore, a step to − 39 mV (100 ms duration) was placed between the hyperpolarizing condition pulse and the test pulse. In WT mice, the current sensitive to the inclusion of the pre-pulse was obtained by graphical subtraction (control—pre-pulse) and peaked within 3–8 ms and completely inactivated with a single time constant of 34.8 ± 2.5 ms (*n* = 15 cells; for the step to + 1 mV; Fig. 7b) thereby revealing the typical kinetic properties of *I*_A_ in VB TC neurons (Huguenard et al. 1991). In TRIP8b^−/−^ mice, the time constant of inactivation was significantly (*p* < 0.05) faster (*τ*_inact_ = 28.0 ± 1.5 ms, *n* = 14; data not shown).

The voltage dependency of activation of *I*_A_ was investigated by analyzing the peak current amplitudes at all test potentials (Fig. 7c) and constructing steady-state activation curves from the normalized conductance values (Fig. 7d). Comparison between genotypes revealed an activation threshold negative to − 70 mV and *V*_h_ values which were significantly (*p* < 0.001) different between WT (*V*_h_ = − 28.0 ± 1.5 mV, *n* = 11 cells) and TRIP8b^−/−^ (*V*_*h*_ = − 38.7 ± 2.1 mV, *n* = 11 cells; Fig. 7e) mice. The hyperpolarizing shift in the activation curve lead to an increased availability of I_A_ in TRIP8b^−/−^ mice at subthreshold (− 59 mV; WT: 168.6 ± 23.8 pA, *n* = 16 cells; TRIP8b^−/−^: 359.3 ± 27.5 pA, *n* = 14 cells; *p* < 0.001) but not depolarized membrane potentials (+ 1 mV; WT: 2758.0 ± 283.2 pA, *n* = 16 cells; TRIP8b^−/−^: 3194.8 ± 334.6 pA, *n* = 14 cells).

Next, the steady-state inactivation was investigated by holding neurons at − 69 mV and stepping to different conditioning potentials (− 129 to − 19 mV, 2 s duration, 10 mV increment), before stepping to a constant analyzing test potential of − 9 mV of 100 ms duration (Fig. 7f). Steady-state inactivation curves were constructed from normalized current values. Similar to the results obtained for current activation, *V*_h_ values of steady-state inactivation were significantly more depolarized in WT (*V*_h_ = − 59.9 ± 1.3 mV, *n* = 11 cells) compared to TRIP8b^−/−^ (*V*_*h*_ = − 67.8 ± 1.1 mV, *n* = 11 cells; Fig. 7g) mice. While the more hyperpolarized inactivation in TRIP8b^−/−^ mice may be expected to result in less channels available for activation at a given membrane potential, the ‘‘window current’’ between the activation and inactivation curves revealed more tonically active *I*_A_ near the resting potential in knockout animals (Fig. 7h).

These findings indicate that alterations in *I*_A_ may contribute to the differences in firing pattern found in TRIP8b^−/−^ mice.

### Reduction of burst activity in thalamic network in the absence of TRIP8b

**Fig. 8:**
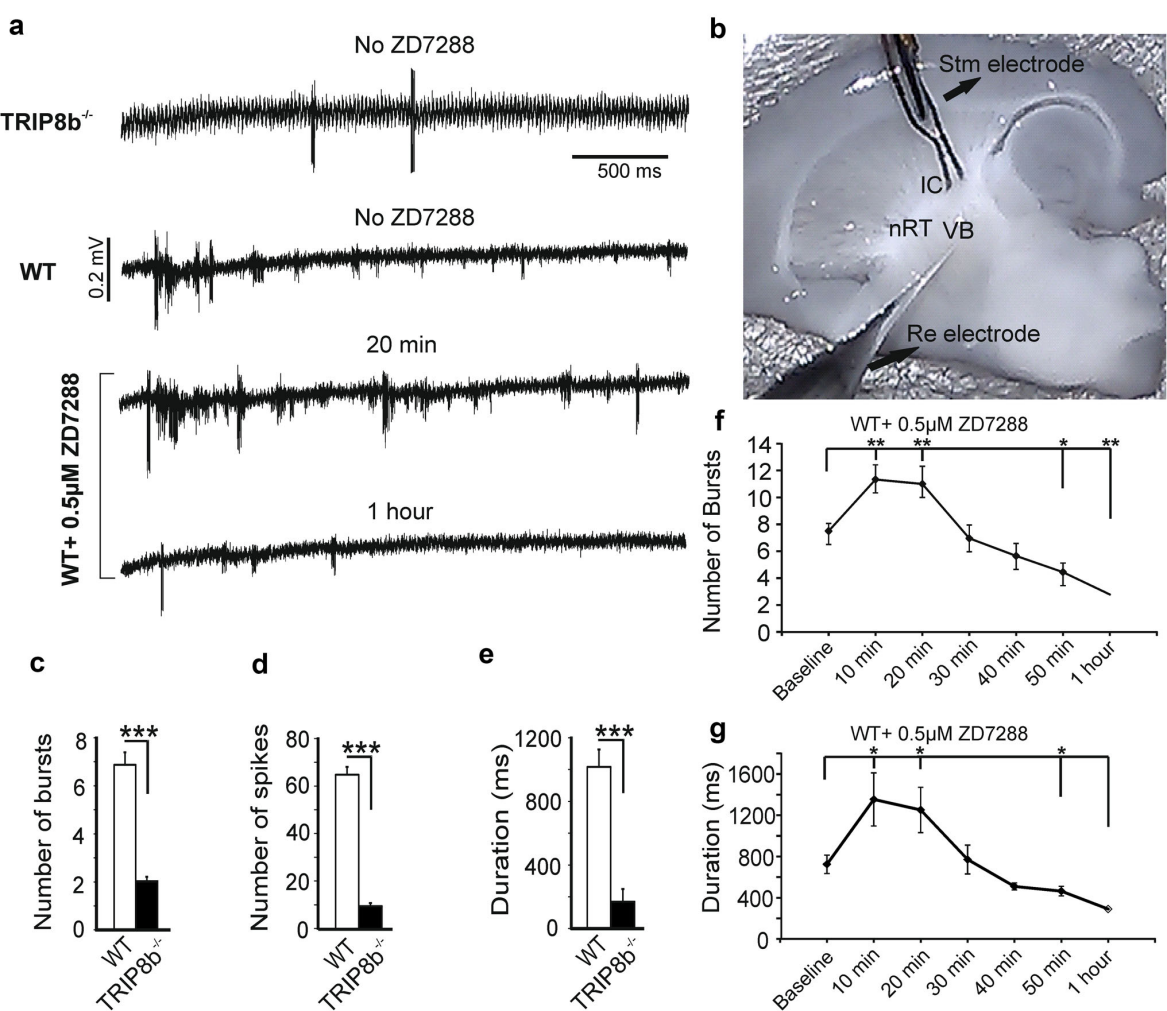
Reduction of oscillatory burst activity in the thalamus of TRIP8b^−/−^ mice. **a** Extracellular field potential recordings of burst activity in the VB complex evoked by stimulation of the IC. TRIP8b^−/−^ mice (*n* = 8 mice) show significantly lower numbers of evoked rhythmic bursts (Student’s *t* tests, *** indicates *p* < 0.001) compared to WT mice (*n* = 8 mice). **b** Image showing the position of recording (Re) and stimulation electrodes (Stm). **c** Bar graph comparing the mean number of bursts in VB complex of TRIP8b^−/−^ and WT mice in response to a single stimulus (Student’s *t* tests, *** indicates *p* < 0.001). **d** Bar graph comparing the total number of spikes during the burst activity between TRIP8b^−/−^ and WT mice (Student’s *t* tests, *** indicates *p* < 0.001). **e** Shorter duration of stimulus-induced burst activity in TRIP8b^−/−^ compared to WT mice (Student’s *t* tests, *** indicates *p* < 0.001). **f** and **g** Bath application of 0.5 µM ZD7288-induced time-dependent effects on intrathalamic network activity (Repeated-measures ANOVAs followed by Student’s *t* tests, *n* = 6 mice, *, **, *** indicate p < 0.05, *p* < 0.01, and *p* < 0.001, respectively. Each data point was compared to the value before ZD7288 application (no ZD7288))

*I*_h_ is an important determinant of oscillatory and rhythmic bursting activity of the thalamic network and has frequently been used as a measure of intrathalamic rhythmicity and possible predisposition for epileptic seizures (Kanyshkova et al. 2012; Kanyshkova et al. 2009; Budde et al. 2005). Intrathalamic network activity is mainly based on reciprocal interactions between TC neurons of dorsal thalamus and GABAergic neurons of nRT and is involved in the generation of different thalamic oscillations such as delta and spindle oscillations. To address the contribution of TRIP8b to evoked thalamic oscillations, dampened oscillatory activity (Fig. 8a) was induced through stimulation of the IC (Fig. 8b) and multiunit recordings were performed in the VB complex (Broicher et al. 2007; Yue and Huguenard 2001). As shown in Fig. 8, compared to WT animals, TRIP8b^−/−^ mice showed a significantly lower number of bursts in response to a single stimulus (TRIP8b^−/−^: 1.4 ± 0.4 bursts; WT: 6.9 ± 0.5 bursts; *n* = 8/8 animals, average of 2–3 slices for each animal, *p* < 0.001; Fig. 8c). In addition, the total number of spikes after stimulation (TRIP8b^−/−^: 9.9 ± 1.3 spikes; WT: 65.2 ± 3.2; *n* = 8/8 animals, average of 2–3 slices for each animal, *p* < 0.001; Fig. 8d) and the duration of the bursting (TRIP8b^−/−^: 169.4 ± 78.7 ms; WT: 1017 ± 109.7 ms; *n* = 8/8 animals, *p* < 0.001; Fig. 8e) were significantly reduced in TRIP8b^−/−^ compared to WT mice. Moreover, the frequency of rhythmic bursting was significantly lower in TRIP8b^−/−^ compared to WT mice (TRIP8b^−/−^: 2.3 ± 0.9 Hz; WT: 7.1 ± 0.5 Hz; *n* = 8/8, *p* < 0.001, data not shown).

To corroborate that reduction of stimulus-induced activity in TRIP8b^−/−^ results from the reduction of *I*_h_, we applied different concentrations of ZD7288 in slices from WT mice (Fig. 8a, f and g). While bath application of 10 µM ZD7288 completely abolished the intrathalamic network activity (data not shown), application of 0.5 µM ZD7288 led to a time-dependent bidirectional effect on thalamic network activity, with a significant initial increase in the number (WT: from 7.5 ± 0.5 to 11.3 ± 1.1 and to 11.0 ± 1.3 after 10 and 20 min of ZD7288 wash in, respectively, *p* < 0.01; Fig. 8a, f) and total duration of rhythmic activity (WT: from 700 ± 90 to 1354 ± 260 ms and to 1250 ± 220 ms after 10 and 20 min of ZD7288 application, *p* < 0.01; Fig. 8g). This effect was followed by a significant decrease in thalamic network activity after 1 h of ZD7288 treatment (number of bursts 2.8 ± 0.7; total duration of the rhythmic activity: 290 ± 70 ms; *p* < 0.05; Fig. 8f, g). The latter value was comparable to rhythmic activity measured in thalamic slices from TRIP8b^−/−^ mice. Moreover, the frequency of rhythmic bursting slowed down over time, i.e., from the theta (7.0 ± 0.5 Hz) to delta frequency range (2.7 ± 0.9 Hz, *p* < 0.01, data not shown).

These results indicate that the level of *I*_h_ dynamically influences the frequency of thalamic network oscillations and that changes found in TRIP8b^−/−^ mice mainly alter the delta frequency activity.

### Mathematical modeling of intrathalamic and thalamocortical network activity

**Fig. 9:**
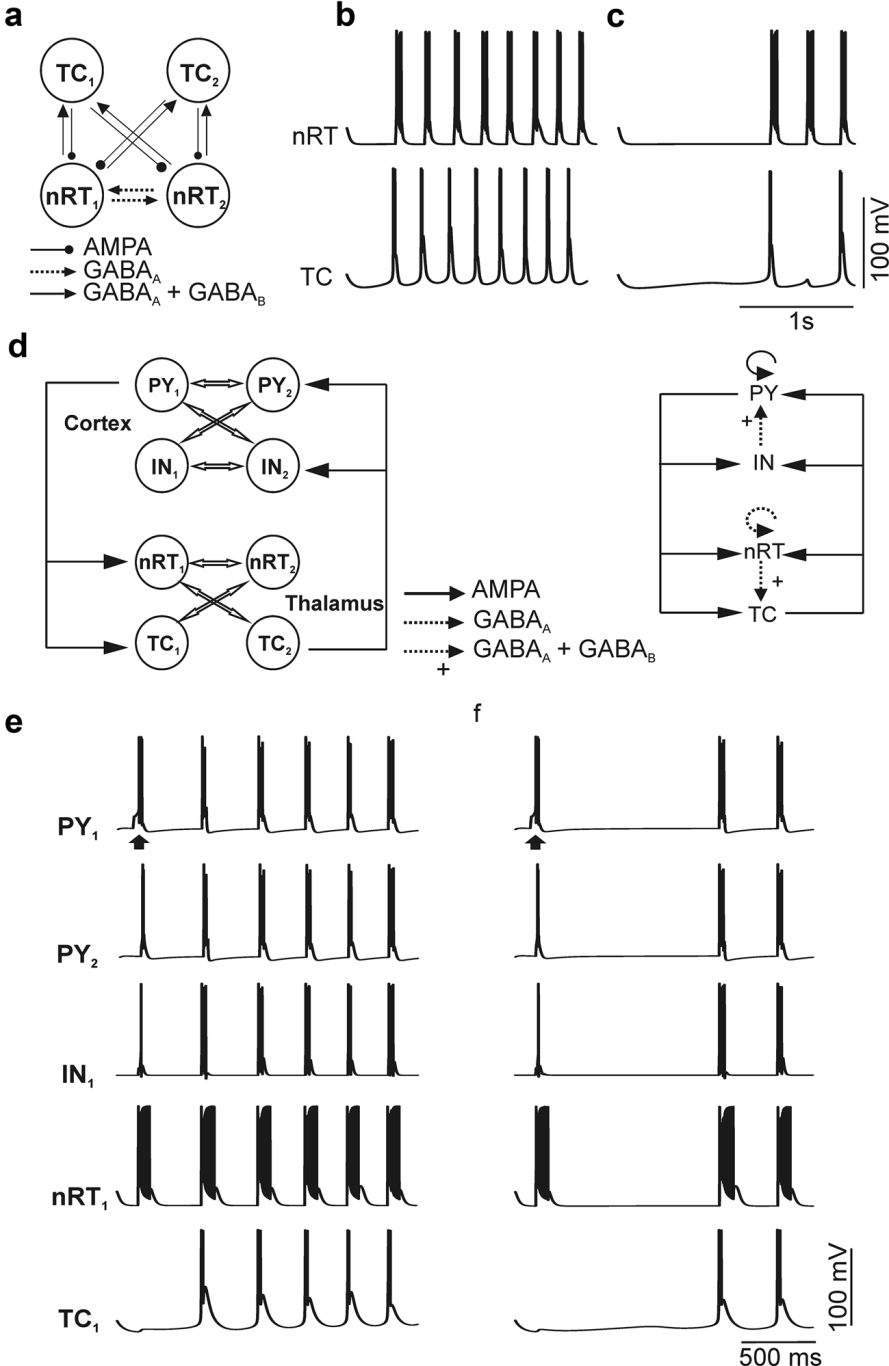
Mathematical modeling of intrathalamic and corticothalamic network activity. **a** The network topology as well as the connection parameters corresponds to Destexhe (Destexhe et al. 1996). In this 4-cell model nRT (reticular thalamic neurons) neurons reciprocally communicate via GABA_A_-mediated connections and project to both TC neurons via GABA_A_ and GABA_B_ signaling. The feedback from TC cells is carried by AMPA receptors to both nRT cells. Since both thalamic relay neurons (TC) as well as both reticular thalamic neurons (nRT) show identical results, only one of each cell type is presented here. At − 70 mV, with *I*_h_ parameters set to the actual values obtained from VB TC cells of WT and TRIP8b^−/−^ mice, the frequency of burst activity (Hz) in the absence of TRIP8b (**c**) is significantly slower compared to WT (**b**) conditions (Student’s *t* test, *p* < 0.05). **d** The network topology as well as the connection parameters correspond to Destexhe (Destexhe et al. 1998). Four cell types were used in this simulation. PY, IN, TC and nRT stand for cortical pyramidal neuron, cortical interneurons, thalamocortical cells and reticular thalamic neurons, respectively. **e** Oscillations were initiated by electrical stimulation of one pyramidal neuron (PY1) indicated by arrows. Actual *I*_h_ parameters obtained from PY and TC neurons of WT (**e**) and TRIP8b^−/−^ (**f**) mice under voltage-clamp conditions were used for the simulation. At − 70 mV, oscillations occurred at a slower frequency range in TRIP8b^−/−^ compared to WT conditions (Student’s *t* test, *p* < 0.05)

Since the horizontal slice preparation allows only a limited number of recurrent activity cycles following electrical stimulation, we assessed how changes in *I*_h_ induced by loss of TRIP8b influence spontaneous sustained rhythmic activity in the thalamocortical network using a mathematical modeling approach. Spontaneous rhythmic bursting was analyzed in a four-cell model which was used for simulation of the intrathalamic network activity (Fig. 9a) and an eight-cell model to simulate thalamocortical network activity (Fig. 9d). Simulation was initiated by releasing the TC neurons from a starting membrane potential (RMP) of − 70 mV. When the simulation was run with *I*_h_ properties determined in WT VB cells, both TC neurons generated spontaneous burst activity with a frequency of 4.2 Hz (*n* = 9 trials; Fig. 9b). *I*_h_ properties in TC neurons were then set to simulate the combinations of *I*_h_ conductance, *V*_0.5_, *k* and activation kinetics observed in TRIP8b^−/−^ neurons (*n* = 10 trials; Fig. 9c) as obtained in voltage-clamp recordings. Since TRIP8b is not expressed in GABAergic neurons of the nRT, the properties of nRT cells were not changed in the model. Compared to WT, the frequency of intrathalamic network oscillations based on parameters obtained from TRIP8b^−/−^ mice was significantly slower (2.5 ± 0.4 Hz, *p* < 0.01). Furthermore, in one run out of ten using TRIP8b^−/−^ parameters, no rhythmic network activity was generated, and in another run just one burst was generated, while all runs with WT parameters resulted in sustained rhythmic activity.

In the eight-cell model (Fig. 9d–f), oscillations were evoked by electrical stimulation of one cortical pyramidal cell (PY1). When the *I*_h_ modules of cortical pyramidal and TC neurons were parameterized with values obtained from WT neurons, the network displayed oscillations with a frequency of about 4 Hz and all cell types discharged in one phase of a cycle. Simulation of thalamocortical network oscillations using *I*_h_ parameters obtained from TRIP8b^−/−^ mice for cortical pyramidal and TC neurons showed significantly slower oscillatory activity in a frequency range of about 2 Hz (*n* = 6 trials for both WT and TRIP8b^−/−^, Student’s *t* test,* p* < 0.01).

These data indicate that the alterations of *I*_h_ induced by the knockout of TRIP8b lead to the slowing of thalamocortical network activity towards delta frequency.

## Discussion

The present study sought to understand the functional role of TRIP8b in the thalamocortical system by combining expression analysis, in vitro and in vivo electrophysiological approaches, in both wild-type (WT) and TRIP8b knockout (TRIP8b^−/−^) mice, as well as computer simulation of thalamocortical activity. TRIP8b^−/−^ mice exhibited reduced expression of HCN channels and *I*_h_ current density leading to hyperpolarized RMP of TC neurons and altered firing properties. Reduced *I*_h_ in the thalamocortical system of TRIP8b^−/−^ mice was associated with altered oscillatory activity and increased delta frequency neuronal activity in horizontal slices, mathematical models, and in animals during episodes of active-wakefulness. TRIP8b^−/−^ mice also exhibited suppression of the normal desynchronization of thalamocortical oscillations at the transition from slow-wave sleep to wakefulness. These findings identify TRIP8b as a necessary molecule which contributes to EEG desynchronization and TRIP8b deficiency as a likely explanation for thalamocortical dysrhythmia, a perturbation of normal thalamocortical activity that has been observed in various neuropsychiatric disorders including schizophrenia and depression (Llinás et al. 1999; Schulman et al. 2011).

### Contribution of TRIP8b to the HCN channel function in TC neurons of different thalamic nuclei

A major function of TRIP8b in the mammalian brain is the regulation of HCN channel trafficking and surface localization (Lewis et al. 2009; Piskorowski et al. 2011; Santoro et al. 2009). In line with previous studies of the hippocampus (Lewis et al. 2011) and VB thalamus (Heuermann et al. 2016), we found a strong downregulation of *I*_*h*_ in TRIP8b^−/−^ TC neurons due to reduced HCN channel protein expression. Here, we extend this finding by demonstrating that TRIP8b upregulates *I*_*h*_ in several functionally different thalamic areas: dLGN, the primary relay for visual information; VB and PO, which form complementary somatosensory pathways with distinct inputs and targets (Kleinfeld and Deschênes 2011); and CM, involved in arousal and attention (Schiff et al. 2013). The fine-tuning of TC neuron properties within these different nuclei which is mandatory to suit their respective roles in the thalamocortical network is influenced by the variations in *I*_h_ density, HCN subunit composition, and specific contribution(s) of TRIP8b. It is interesting to note that TRIP8b is not expressed in GABAergic thalamic neurons, namely nRT neurons and local circuit interneurons (Heuermann et al. 2016; present study, see Supplemental Figure 5 a–c). The preserved *I*_h_ in these cells may partially explain the infrequent absence seizures (Heuermann et al. 2016) and short bursts of activity in our LFP recordings in TRIP8b^−/−^ mice, as compared to the severe epileptic phenotype of HCN2 knockouts (Ludwig et al. 2003).

HCN channels are partially open at rest and thus critically contribute to the RMP and *R*_in_ in TC neurons. Knockout of HCN2 (Ludwig et al. 2003; Huang et al. 2009; Budde et al. 2012; Meuth et al. 2006), HCN4 (Budde et al. 2012), or pharmacological blockade of I_h_ (Leist et al. 2016) shifts the RMP to more hyperpolarized potentials. Similarly, we found that downregulating *I*_h_ by knocking out TRIP8b produced a robust − 11 mV shift in RMP in TC neurons, accompanied by a 45% increase in *R*_in_. We further demonstrated that these changes in membrane properties have pronounced effects on the firing properties of TC neurons in TRIP8b^−/−^ mice, increasing the propensity for burst firing in response to depolarizing inputs but preventing rebound bursts following injection of hyperpolarizing currents. Due to the hyperpolarized RMP and lack of *I*_h_-dependent afterdepolarizing potential (see Fig. 5, right upper panel) that occurs in WT cells, TRIP8b^−/−^ neurons remain below the threshold for activating T-type Ca^2+^ channels, which are responsible for the generation of the rebound burst. Notably, injection of hyperpolarizing current from a holding potential of − 60 mV (which is above the threshold for activating T-type Ca^2+^ channels) resulted in the reappearance of rebound bursts in TRIP8b^−/−^ TC cells, suggesting that neuromodulatory influences could potentially restore this capability in vivo. Rebound bursting is important for different physiological and pathophysiological oscillatory activity within the thalamocortical network, particularly sleep spindles, delta oscillations and perhaps also spike-wave discharges (SWDs), mediated by reciprocal innervation between nRT and TC neurons (Steriade 2003).

In addition to alterations in burst firing, TRIP8b^−/−^ TC neurons showed a significant reduction in tonic AP firing upon injection of depolarizing current steps compared to WT animals. Extracellular application of 10 µM ZD7288 to WT TC neurons had a similar effect, as has been observed before in thalamic, hypothalamic and DRG neurons (Leist et al. 2016; Momin et al. 2008; Cai et al. 2012). This is in contrast to results in cortical and hippocampal pyramidal neurons, in which loss of *I*_*h*_ enhances AP firing (Huang et al. 2009; Lewis et al. 2011). This discrepancy may result from differences in HCN subunit expression and subcellular localization, causing the tonic depolarizing influence of *I*_h_ to be more important in some neurons (e.g., TC neurons), whereas the effect on *R*_in_ dominates in others (pyramidal neurons). Another notable finding is that the reduction in the number of APs induced by ZD7288 was less extensive compared to TRIP8b knockout animals, even after restoring the RMP to − 60 mV with DC current injection. These results indicate that there might be additional mechanisms underlying the reduction of AP firing in TC cells of TRIP8b^−/−^ mice. Possible mechanisms may be related to the decreased cAMP levels and/or alterations in K^+^ channel function in TRIP8b^−/−^ mice. Since increases in cAMP enhance tonic firing in rodent TC neurons (Ehling et al. 2013) the lower basal cAMP level in TRIP8b^−/−^ mice brain compared to WT might contribute to the reduction of AP firing in TC cells in these animals. Another possible candidate is changes in Kv1.2 channels, which have been shown to increase their amplitude upon co-expression with TRIP8b (Santoro et al. 2009). Since Kv1.2 channels are expressed in TC neurons (Decher et al. 2010) and Kv1.2-deficient mice show decreased AP firing in neurons (Robinson et al. 2003), our results are in agreement with a reduction of current through Kv1.2 channels. Although no direct interaction was found between TRIP8b and channels underlying *I*_*A*_ in central neuron (Santoro et al. 2009), this fast transient K^+^ current is a potential candidate since it is modulated by cAMP and known to shape bursting and tonic firing of TC neurons (Pape et al. 1994; Kanyshkova et al. 2011). The voltage-dependent properties of *I*_A_ as described in the present study are well within the range of parameters found for activation (V_h_: − 14 to − 37 mV), inactivation (V_h_: − 65 to − 82 mV) and current kinetics (*τ*_inact_: 6 to 96 ms) of fast transient K^+^ currents in TC neurons in different thalamic nuclei and species (Huguenard et al. 1991; Budde et al. 1992; Kanyshkova et al. 2011; McCormick 1991; Noh et al. 2011). In addition, a slow inactivating *I*_A_ component generating a large window current was described (McCormick 1991). The firing properties of TC neurons are influenced by *I*_A_ in several ways. The presence of a large K^+^ current that generates a window current and is active at rest is expected to influence the RMP, bursting and tonic firing. Indeed computer modeling as well as pharmacological block by 4-amino pyridine (4-AP) revealed the hyperpolarizing influence of *I*_A_ on the RMP (McCormick 1991; Amarillo et al. 2014). Furthermore, electrophysiological recordings and computer simulations have demonstrated that *I*_A_ controls the generation, amplitude and duration of a LTS by functionally counteracting *I*_T_ (Huguenard et al. 1991; Pape et al. 1994; Amarillo et al. 2014; Gutierrez et al. 2001; Noh et al. 2010). Since *I*_A_ is active during repetitive bursting at the initial depolarizing phase of each cycle, the magnitude of the current influences the periodicity (Amarillo et al. 2014). While bursting requires hyperpolarized levels of the membrane potential, *I*_A_ also affects tonic firing from more depolarized potentials when *I*_T_ is inactivated. Since depolarizing inputs have to overcome the hyperpolarizing influence of the inactivating *I*_A_, the onset of firing is delayed thereby limiting the number of induced APs (McCormick 1991; Budde et al. 1992; Kanyshkova et al. 2011). Wash in of 4-AP abolishes the delayed onset of firing and increases the half width of single APs (Budde et al. 1992; Kanyshkova et al. 2011). While the latter decreases tonic firing frequencies, the total number of APs elicited by a given depolarizing pulse is increased, thus demonstrating the limitation of AP firing by *I*_A_. This view is corroborated by findings from different neuronal cell types where receptor stimulation-dependent activation and inhibition of I_A_ reduces and increases the rate of firing APs, respectively (Kloppenburg et al. 1999; Pitra and Stern 2017). In a similar way the knock down of Kv4.1 channels increased neuronal firing rates (Hermanstyne et al. 2017).

Since several members of different K_V_ channel subfamilies including K_V_1.4, K_V_3.3, K_V_3.4, K_V_4.1-4.3 reveal typical *I*_*A*_ properties, the molecular basis of fast transient K^+^ currents is complex (Song 2002). This complexity is even increased by the formation of multi-protein ion channel complexes that underlie native currents. In case of *I*_*A*_, the K_V_ channel-interacting proteins (KChIPs) and dipeptidyl peptidase-like proteins (DPLPs) interact with the pore-forming *α*-subunits (Jerng and Pfaffinger 2014). Based on expression studies and analysis of native current, combinations of K_V_4.2/K_V_4.3, KChIP3/KChIP4, and DPP6/DPP10 may contribute to I_A_ in thalamic TC neurons (Serôdio and Rudy 1998; Rhodes et al. 2004; Xiong et al. 2004; Wang et al. 2014; Kanyshkova et al. 2011).

Based on the functional properties and expression profile of transient K^+^ channels discussed above, a number of alterations found in TRIP8b^−/−^ mice may be attributed to the reduction in cAMP and consequent changes in *I*_A_. Channels based on the combination of K_V_4.2 and KChiP3 have been shown to undergo a rightward shift in the activation curve and slowing of inactivation kinetics following cAMP-dependent modulation (Schrader et al. 2002). Therefore, the leftward shift in the *I*_A_ activation curve and the faster inactivation kinetics are in line with the decreased cAMP levels found in TRIP8b^−/−^ mice. Additional interactions with DPP6 may result in the hyperpolarizing shift of the inactivation curve in TRIP8b^−/−^ mice (Jerng and Pfaffinger 2014). Together these changes enlarge the window current, increase the tonically active *I*_A_ near the RMP and increase the voltage-activated *I*_A_ close to the AP threshold (Kloppenburg et al. 1999). In combination with the reduction in *I*_h_, increased tonic *I*_A_ hyperpolarizes the RMP (Amarillo et al. 2014) and reduces tonic firing by delaying the onset of firing and reducing the number of evoked APs. When an LTS is evoked in TC neurons of TRIP8b^−/−^ mice the enlarged *I*_A_ is shaping the time course by contributing to reduced amplitude and duration. In addition, the combined changes in *I*_A_ and *I*_h_ may help to sustain oscillatory LTS generation at low frequency. While a decreased *I*_h_ is expected to promote oscillations, conditions of imposed hyperpolarization may further facilitate oscillatory activity (Amarillo et al. 2014). With respect to thalamocortical oscillations, it is interesting to note that the block of I_A_ by 4-AP elicited spontaneous field potential activity in thalamocortical slices in vitro (D’Arcangelo et al. 2002). While sequences of fast (10–16 Hz) and slower (5–9 Hz) field potential bursts were recorded in WAG/Rij rats, a rodent absence epilepsy model (van Luijtelaar and Zobeiri 2014), only fast oscillations were seen in non-epileptic control rats. SWDs in the frequency range of 7–9 Hz are characteristic for a number of rodent absence epilepsy models. Therefore, the increased *I*_A_ window current may hinder the transition from slow oscillations to faster epilepsy-related SWDs and wake-related rhythms.

### TRIP8b regulates the cAMP sensitivity of *I*_h_ in TC neurons

In addition to affecting surface expression, TRIP8b regulates gating and cAMP-sensitivity of HCN channels (Lewis et al. 2009; Santoro et al. 2009; Zolles et al. 2009; Hu et al. 2013; Saponaro et al. 2014). Previous in vitro studies have demonstrated that TRIP8b is an allosteric inhibitor for cAMP binding (Hu et al. 2013; Saponaro et al. 2014; Deberg et al. 2015), and interaction of TRIP8b with the CNBD antagonizes the effects of cAMP on channel gating (i.e., induces a negative shift in *V*_0.5_ and slows down activation kinetics). In accordance with the antagonizing effects of TRIP8b on cAMP and similar to a previous report (Heuermann et al. 2016), we found a significant increase in the sensitivity and efficacy of cAMP in TC neurons in TRIP8b^−/−^ mice compared to WT animals. Unexpectedly, *V*_0.5_ values of *I*_*h*_ in TRIP8b^−/−^ TC neurons in the present study were significantly more hyperpolarized compared to WT cells, and channel activation was slower. Since HCN channels in rodent TC neurons have been shown to be controlled by basal cAMP production (Wainger et al. 2001; Pape 1996; Kanyshkova et al. 2009), the negative shift in the activation curve of *I*_h_ and its slower activation kinetics pointed to possible changes in cAMP signaling in TRIP8b^−/−^ mice. In fact, reduced cAMP levels were determined in brain tissue samples from TRIP8b^−/−^ mice compared to WT animals. Along these lines, the adenylyl cyclase blocker SQ22536 and the A1 receptor agonist CCPA both shifted the activation curve of *I*_h_ to more hyperpolarized potentials in WT neurons. Of note, Heuermann et al. (2016) found no difference in basal *V*_0.5_ values between WT and TRIP8b^−/−^ TC neurons. An intriguing possibility to explain this discrepancy may be that the coronal slices used here better preserve ascending neuromodulatory inputs (e.g., hypothalamic histaminergic projections) that were severed in horizontal slices used in the previous study. If this were the case, it might obscure differences in basal cAMP levels and thus *V*_0.5_ during slice recordings.

### TRIP8b regulates *I*_h_ in cortical pyramidal neurons

Previous studies demonstrated a high expression level of TRIP8b in layer V cortical pyramidal neurons with an expression gradient similar to HCN1 channels (Santoro et al. 2004). In these cells, TRIP8b is responsible for the trafficking of HCN1 subunits to the cell membrane. In TRIP8b^−/−^ mice, the lack of TRIP8b in cortical pyramidal neurons resulted in a reduction in the expression of HCN1/2 subunits in the somatosensory cortex whereas the mRNA levels were not affected. In accordance with these results, layer V and VI cortical pyramidal neurons of TRIP8b^−/−^ mice showed a significantly lower *I*_h_ density compared to WT animals. The reduction of *I*_h_ in cortex is responsible for an increased *R*_in_ and a concomitant increase in dendritic excitability in these neurons. In fact, the downregulation of *I*_h_ in cortical pyramidal neurons has been reported in several types of pathophysiological conditions including absence epilepsy (Huang et al. 2009; Phillips et al. 2014; Heuermann et al. 2016; Kole et al. 2007). However, in contrast to the previous study by Heuermann et al. (2016), our in vivo LFP recordings from the somatosensory cortex of TRIP8b^−/−^ mice revealed only sporadic short (< 1 s) epileptiform-like activity in a small number of animals. No definite reason for the differences between the two studies can be named except a potential genetic drift, especially in a small founder colony (Sade et al. 2014). It has been noted before that differences in epilepsy severity between different colonies of GEARS rats that were derived from the same founder colony may be based on environmental conditions and/or genetic drift (Powell et al. 2014). Early life environmental experience can influence the frequency of SWD occurrence in adulthood (van Luijtelaar and Sitnikova 2006). In WAG/Rij rats, maternal deprivation and neonatal handling from postnatal day 1 to 21 reduced the number of SWDs in adulthood (4.5 months of age) with about 35%, while the mean duration was not affected (Schridde et al. 2006). In addition, introducing an enriched environment (for 2 months from 1 to 3 months of age) was found to have no effect on the number of animals with SWDs and no effect on the number of SWDs, only on the mean duration of SWDs (Schridde and van Luijtelaar 2004). In addition, cross-fostered WAG/Rij pups which were raised by Wistar mothers showed fewer SWDs compared to the condition in which they were cross-fostered within the WAG/Rij strain (Sitnikova et al. 2016). Altogether, these data suggest that environmental factors play a role in shaping the occurrence of SWDs in WAG/Rij rats, but not to a very large extent and especially not for the number of animals that show SWDs. Earlier manipulations (postnatal) seem to play a larger effect than post-weaning manipulations regarding the number and mean duration of SWDs. After comparing the environmental conditions in which the two groups of TRIP8b^−/−^ mice were raised, we found some minor differences (e.g., differences in light/dark cycle and hygienic procedures, but not general handling) that might to some degree affect the occurrence of SWDs in our TRIP8b^−/−^ mice colony.

### The role of TRIP8b in regulation of thalamocortical oscillations and modulation of delta oscillations

In the present study, we demonstrated that the dysregulation of *I*_h_ in the thalamocortical system of TRIP8b^−/−^ mice is associated with altered thalamocortical oscillations, revealing a significant increase in slow oscillations in the delta frequency range during episodes of active-wakefulness and reduced desynchronization of the EEG during transitions from slow-wave sleep to active-wakefulness. Considering that the behavior during active-wakefulness of TRIP8b^−/−^ mice did not appear qualitatively and quantitatively (as was established in the open field test (Lewis et al. 2011)) different from that of WT, it is safe to exclude that the differences in EEG between the mice strains is due to differences in overt behavior. More general, the appearance of prominent delta waves during wake is associated with both physiological and pathological conditions. In the cognitive domain, increased delta frequency oscillations are implicated in attention and salience detection and subliminal perception (Knyazev 2012). However, increased baseline delta power has also been associated with a range of neurological disorders including Alzheimer’s disease (Jeong 2004; Babiloni et al. 2009), and schizophrenia (Boutros et al. 2008). Interestingly, while TRIP8b^−/−^ mice were largely phenotypically normal during initial behavioral screening (Lewis et al. 2011), they did exhibit significantly impaired nest building, an established endophenotype for schizophrenia (Amann et al. 2010), raising the possibility that reduced thalamocortical HCN channel function may recapitulate some features of this disorder.

Several neurotransmitter systems contribute to promotion of sleep and wakefulness and regulation of cortical activation during different behavioral states. Both thalamus and neocortex receive a large number of cholinergic projections from pontine and midbrain reticular formation, as well as cholinergic (and non-cholinergic) inputs from nuclei in the basal forebrain (BF) activating system (Buzsaki et al. 1988). These cholinergic projections play an important role in the desynchronized EEG typical for wakefulness and REM sleep (Wikler 1952; Steriade et al. 1990). In addition, the serotonergic neurons of the dorsal raphe (DR) and noradrenergic neurons of the locus coeruleus (LC) contribute to both pathways and directly innervate cortical neurons (Brown et al. 2012). A number of observation point to a limited contribution of either TRIP8b or HCN channels to the function of these activating systems, thereby lowering the possibility that changes in the EEG of TRIP8b^−/−^ mice are related to alterations in these areas. In comparison to cortical and thalamic areas, the expression of TRIP8b was low in WT and expression of HCN2 was not altered following TRIP8b knock out in the ascending brainstem-activating system (see Supplemental Figures 10, 11). In addition, electrophysiological characterization of the cholinergic neurons of the BF and the locus coeruleus revealed only very little time-dependent anomalous rectification (Unal et al. 2012; Alreja and Aghajanian 1991; Hedrick and Waters 2010). Nevertheless, a role of activating systems in the aberrant waking EEG pattern in TRIP8b^−/−^ mice cannot be ruled out by the experiments presented here.

Several previous studies have established the importance of both thalamic and cortical I_h_ in generating network oscillations, particularly delta and spindle oscillations during slow-wave sleep (Steriade et al. 1993; Steriade and Deschenes 1984; Crunelli et al. 2015; Kanyshkova et al. 2009). *I*_h_ in cortical pyramidal neurons helps to establish the subthreshold resonance frequency, typically in the theta band, which amplifies oscillations in this frequency range (Hu et al. 2002; Stark et al. 2013; Wahl-Schott and Biel 2009). Downregulation of *I*_h_ shifts the resonant frequency from the theta to delta range both within individual pyramidal neurons (Karameh et al. 2006; Stadler et al. 2014) and at the network level (Schmidt et al. 2016). We, therefore, conclude that the significant reduction of *I*_h_ in cortical pyramidal neurons of TRIP8b^−/−^ mice alters the preference of the cortical network in favor of slower oscillations.

*I*_h_ also contributes to delta rhythmicity at the level of the thalamus by promoting cycles of rebound bursts in reciprocally connected TC and nRT neurons (Crunelli et al. 2015; Steriade et al. 1993; Llinas and Steriade 2006; Steriade 2003; Timofeev and Bazhenov 2005; Kanyshkova et al. 2009; Meuth et al. 2003). Delta oscillations typical for slow-wave sleep occur due to hyperpolarization of TC neurons as sleep deepens, increasing the propensity for bursting (Steriade 2003; Llinas and Steriade 2006). Therefore, one might speculate that the hyperpolarized RMP of TC neurons in TRIP8b mice^−/−^, coupled with a cortical network that resonates at delta frequencies, favors generation of thalamocortical delta oscillations during active-wakefulness. However, impaired rebound bursting in these cells would prevent reverberant, runaway delta activity in the form of SWDs. In line with this hypothesis, our LFP recordings of intrathalamic network activity showed slowed inter-burst frequency in TRIP8b^−/−^ mice and a reduction in overall oscillatory activity. Interestingly, a low concentration of ZD7288 (0.5 µM) generated time-dependent effects, with an increase in rhythmic burst activity during the first 20 min after application followed by a significant decrease in rhythmic burst activity towards the end of recording that mimics the TRIP8b^−/−^ condition. These findings suggest that the level of hyperpolarization and availability of *I*_h_ in thalamic neurons is an important determinant of the behavior of the thalamic network. Slight hyperpolarization is able to increase the rhythmic burst activity in thalamus (as may be the case in the epileptic HCN2^−/−^ mouse), while further hyperpolarization (e.g., TRIP8b^−/−^ TC neurons, with downregulation of both HCN2 and HCN4) can abolish or significantly slow down the intrathalamic network oscillations (Yue and Huguenard 2001). Slowing of oscillatory activity was also obtained after introducing the conductance levels, *V*_0.5_ values and activation kinetics of *I*_h_ obtained from TRIP8b^−/−^ TC cells to thalamocortical network models (Destexhe et al. 1996). Further analyses with similar models will be an excellent tool for probing the graded effects of *I*_h_ within thalamocortical networks, and for guiding future experiments to understand the dynamic roles of HCN channels in modulating both physiological and pathophysiological brain states.

## Conclusion

Here, we demonstrated an increase in delta rhythmicity within thalamocortical networks of TRIP8b^−/−^ mice during episodes of active-wakefulness resulting from reduced *I*_h_ (in combination with increased availability of *I*_A_) and consequently altered firing properties of TC neurons. Our results are consistent with a previous analysis of *I*_h_ in thalamic and cortical neurons of the TRIP8b^−/−^ mouse (Heuermann et al. 2016). Overall, the results obtained from TRIP8b^−/−^ mice point to the importance of TRIP8b as a brain-specific HCN channel auxiliary subunit that regulates cellular and network oscillations. The interaction between TRIP8b and HCN channels seems to be necessary to prevent the occurrence of increased delta activity during wakefulness (thalamocortical dysrhythmia) thereby suggesting TRIP8b as a promising drug target (Han et al. 2015).

## Electronic supplementary material

Below is the link to the electronic supplementary material.

Supplementary material 1 (PDF 62 kb)

Supplementary material 2 (PDF 12468 kb)
