# Supplementary material for: Modulation of thalamocortical oscillations by TRIP8b, an auxiliary subunit for HCN channels
Source: Brain Struct Funct. 2017 Nov 22;223(3):1537–64. doi: 10.1007/s00429-017-1559-z (PMC5869905; doi:10.1007/s00429-017-1559-z)
Supplement: Supplementary file 1 — Supplementary material 1 (PDF 62 kb) [file 429_2017_1559_MOESM1_ESM.pdf]

Supplemental Table.1 I_h activation kinetics in TRIP8b$^{-/-}$ and wildtype thalamus.

Time constant of I_h activation	Regions	WT	TRIP8b$^{-/-}$	Significance
Fast component T_1 (ms)	VB	121.5 ± 4.5	179.0 ± 8.8	p<0.01
	PO	185.6 ± 8.7	226.4 ± 18.7	p<0.01
	CM	157.8 ± 8.3	175.6 ± 15.2	NS
	dLGN	160.9 ± 6.5	205.5 ± 17.8	p<0.05
Slow component T_2 (ms)	VB	587.6 ± 3.6	934.1 ± 55.4	p<0.001
	PO	736.6 ± 35.1	1421.5 ± 215.8	p<0.01
	CM	672.0 ± 47.9	842.9 ± 48.5	p<0.05
	dLGN	880.2 ± 61.8	1457.5 ± 237.7	p<0.05

*NS means not significant
