# Supplementary material for: Modulation of thalamocortical oscillations by TRIP8b, an auxiliary subunit for HCN channels
Source: Brain Struct Funct. 2017 Nov 22;223(3):1537–64. doi: 10.1007/s00429-017-1559-z (PMC5869905; doi:10.1007/s00429-017-1559-z)
Supplement: Supplementary file 2 — Supplementary material 2 (PDF 12468 kb) [file 429_2017_1559_MOESM2_ESM.pdf]

Modulation of thalamocortical oscillations by TRIP8b, an auxiliary subunit for HCN channels

Mehrnoush Zobeiri[1], Rahul Chaudhary[1], Maia Datunashvili[1], Robert J. Heuermann[2], Annika Lüttjohann[1], Venu Narayanan[3], Sabine Balfanz[4], Patrick Meuth[1], Dane M. Chetkovich[2], Hans-Christian Pape[1], Arnd Baumann[4] , Gilles van Luijtelaar[5] ,Thomas Budde[1]

[1]Institut für Physiologie I, Westfälische Wilhelms-Universität, 48149 Münster, Germany

[2]Davee Department of Neurology and Clinical Neurosciences and Department of Physiology, Feinberg School of Medicine, Northwestern University, 60611Chicago, USA

[3]Department of Neurology and Institute of Translational Neurology, Westfälische Wilhelms-Universität, 48149 Münster, Germany

[4]Institute of Complex Systems, Zelluläre Biophysik (ICS-4), Forschungszentrum Jülich, 52425 Jülich, Germany

[5]Donders Centre for Cognition, Radboud University, 6500 Nijmegen, the Netherlands

Correspondence should be addressed to:

Mehrnoush Zobeiri

E-Mail: zobeiri@uni-muenster.de

Telephone number: +49-251-8355566

Thomas Budde

E-Mail: tbudde@uni-muenster.de

Telephone number: +49-251-8355533

Acknowledgements

The authors thank Elke Naß, Alexandra Markovic, Katrin Foraita, Julia Schröer and Svetlana Kiesling for excellent technical assistance. This work has been supported by Deutsches Forschungsgemeinschaft (DFG, BU 1019/15-1), Interdisziplinäres Zentrum für Klinische Forschung Münster (IZKF, Bud3/001/16) and National Institutes of Health (NIH) grants NS059934 and GM008152. Rahul Chaudhary and Maia Datunashvili were DAAD fellows. This work was done in partial fulfillment of the Ph.D. thesis of Mehrnoush Zobeiri.

Supplemental Fig.1

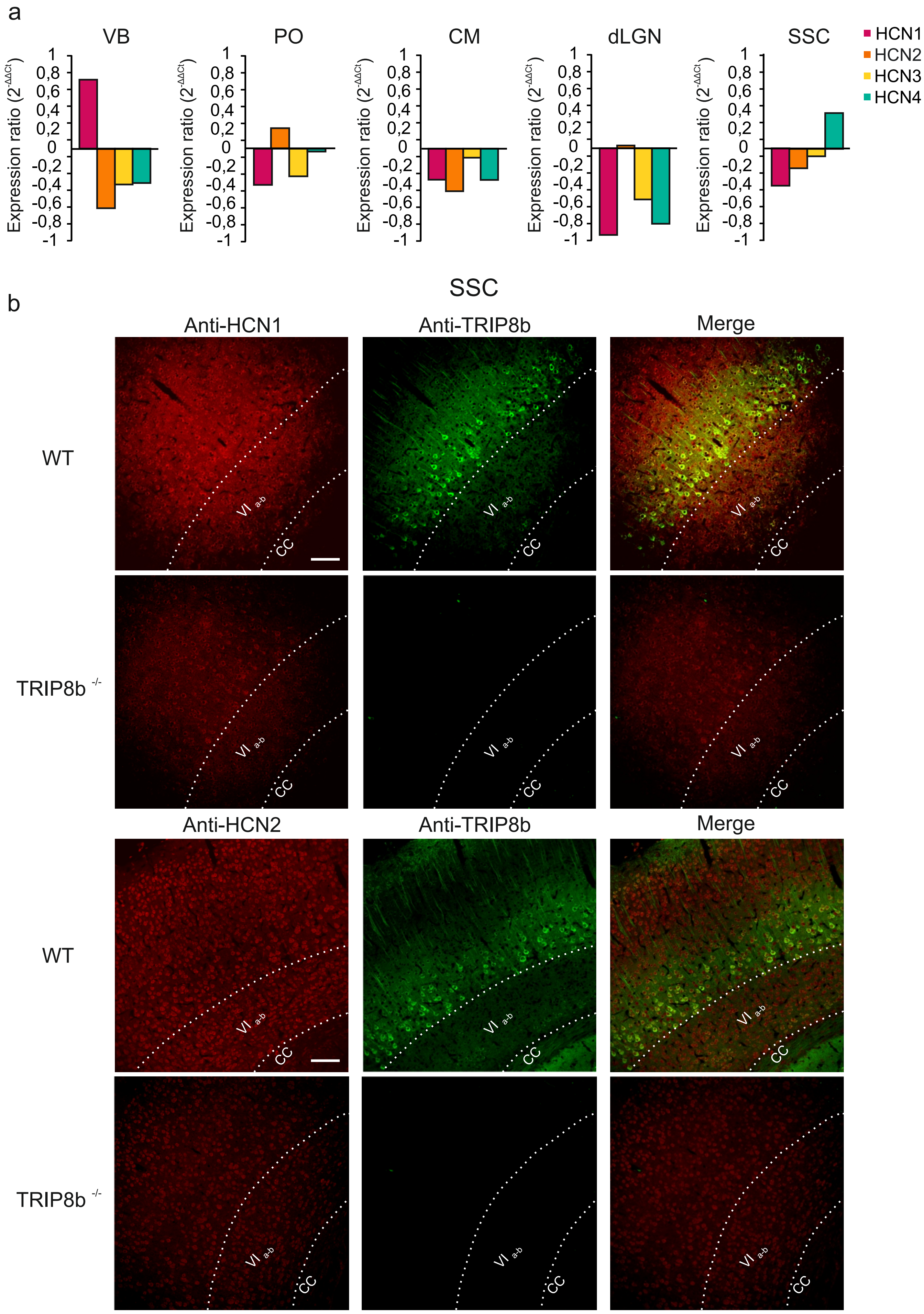

Supplemental Fig.2

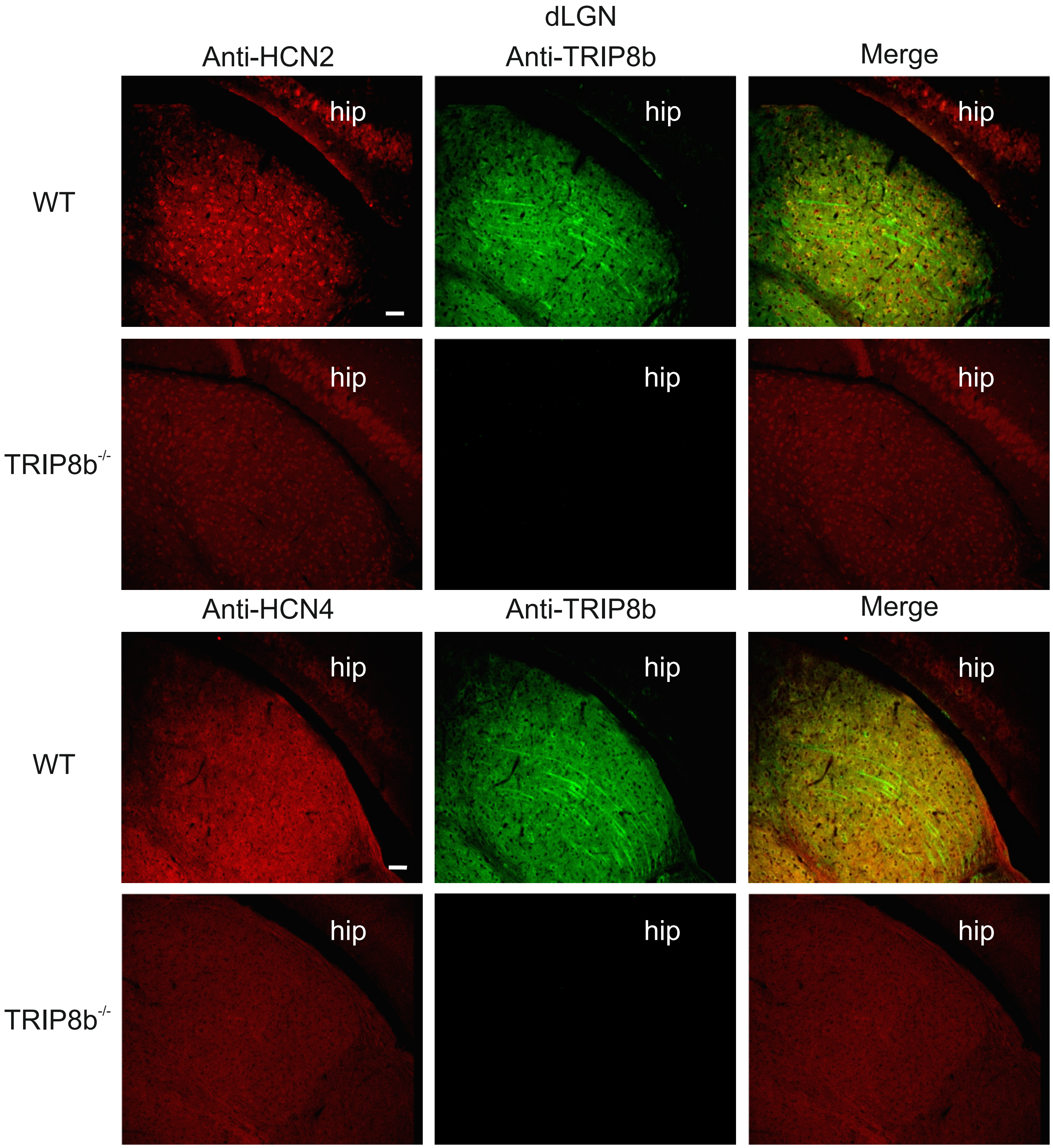

Supplemental Fig.3

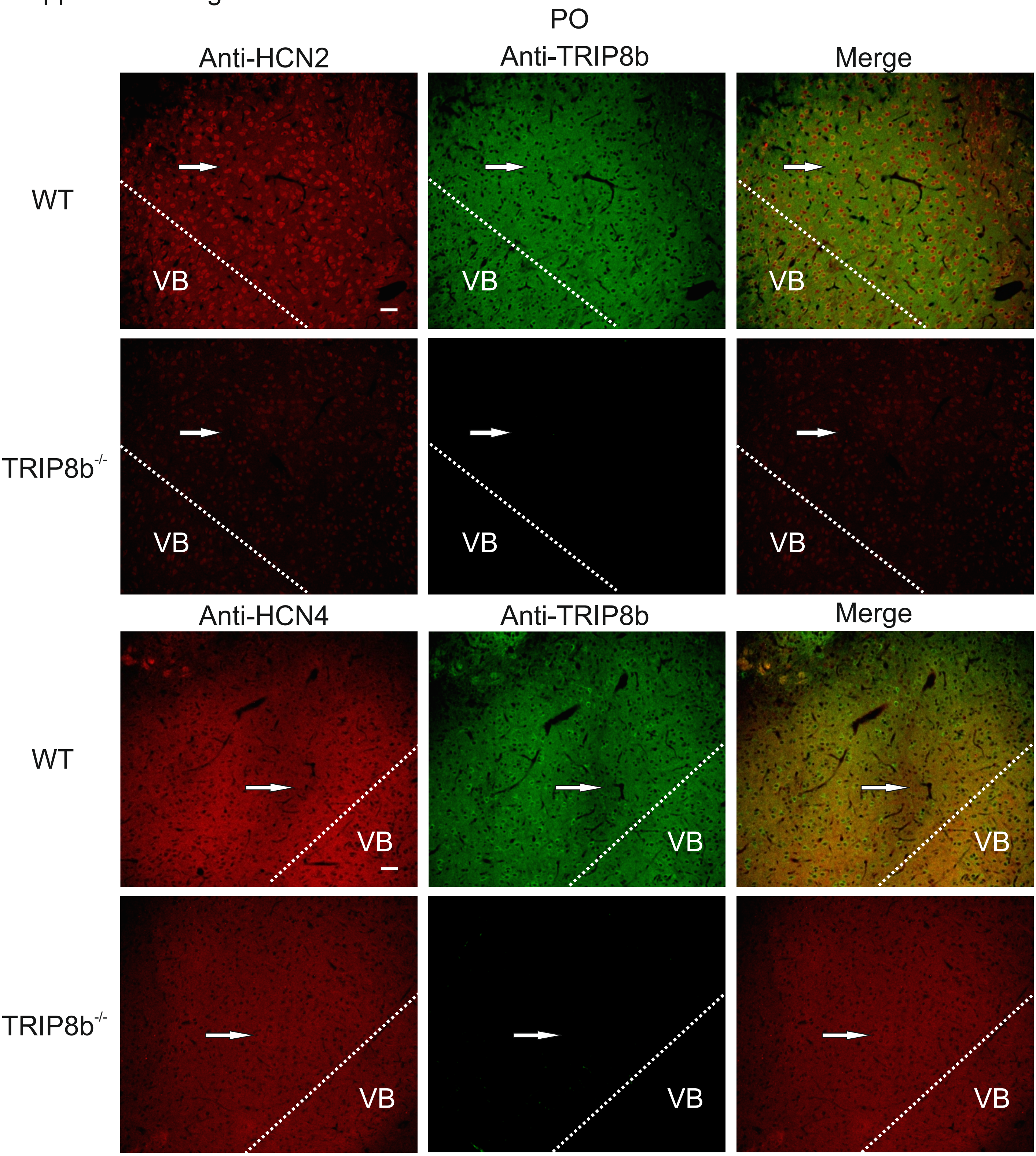

Supplemental Fig.4

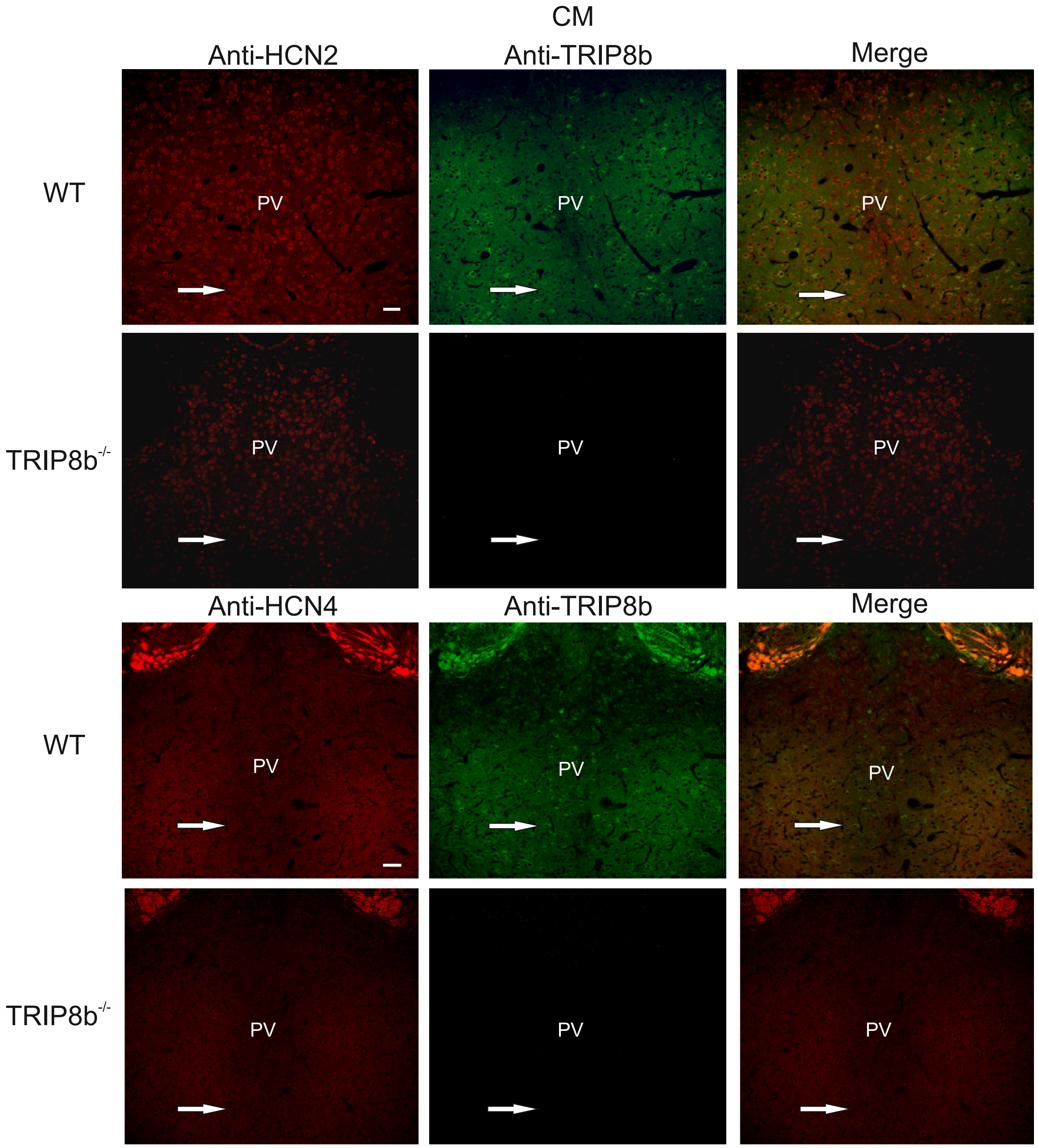

Supplemental Fig.5

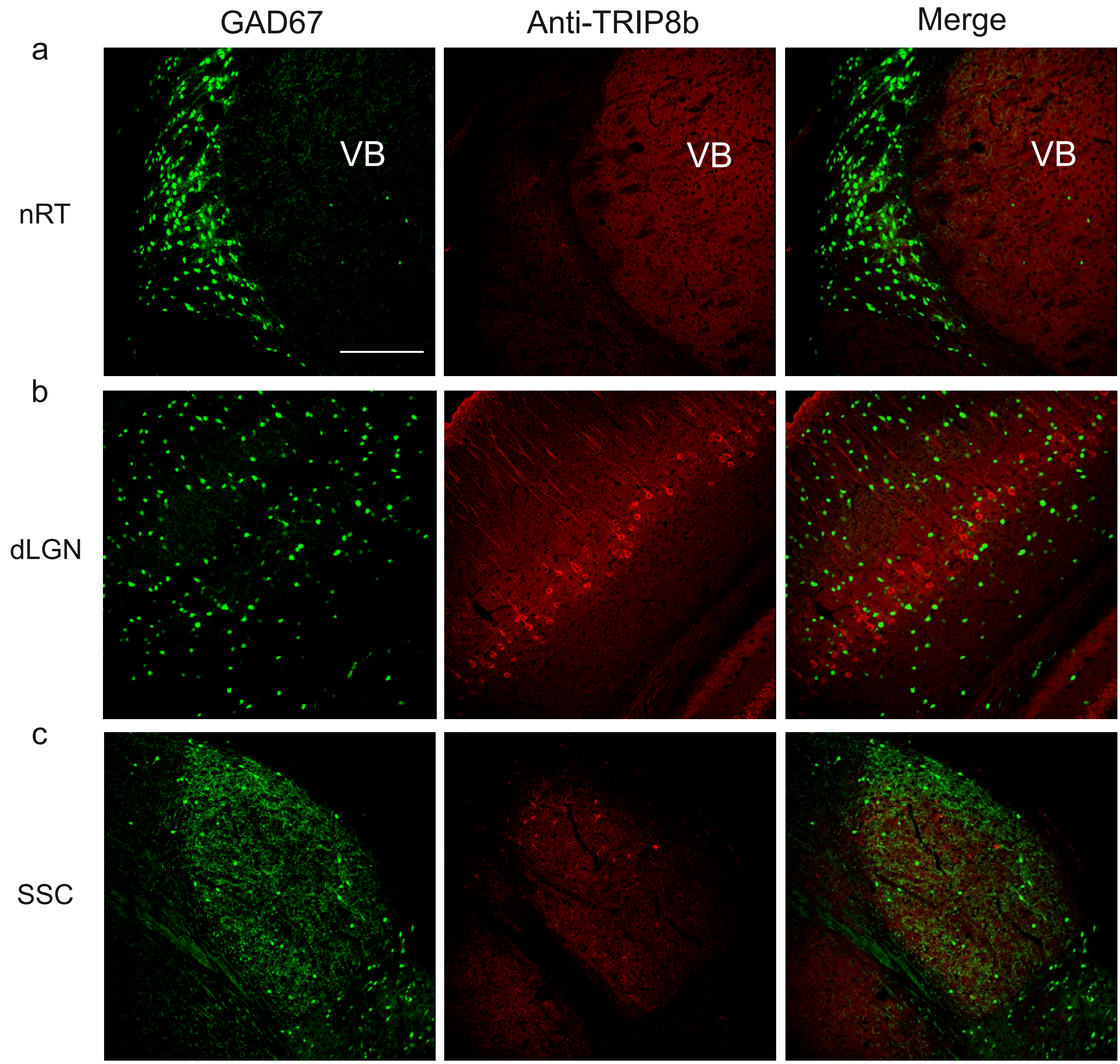

Supplemental Fig.6

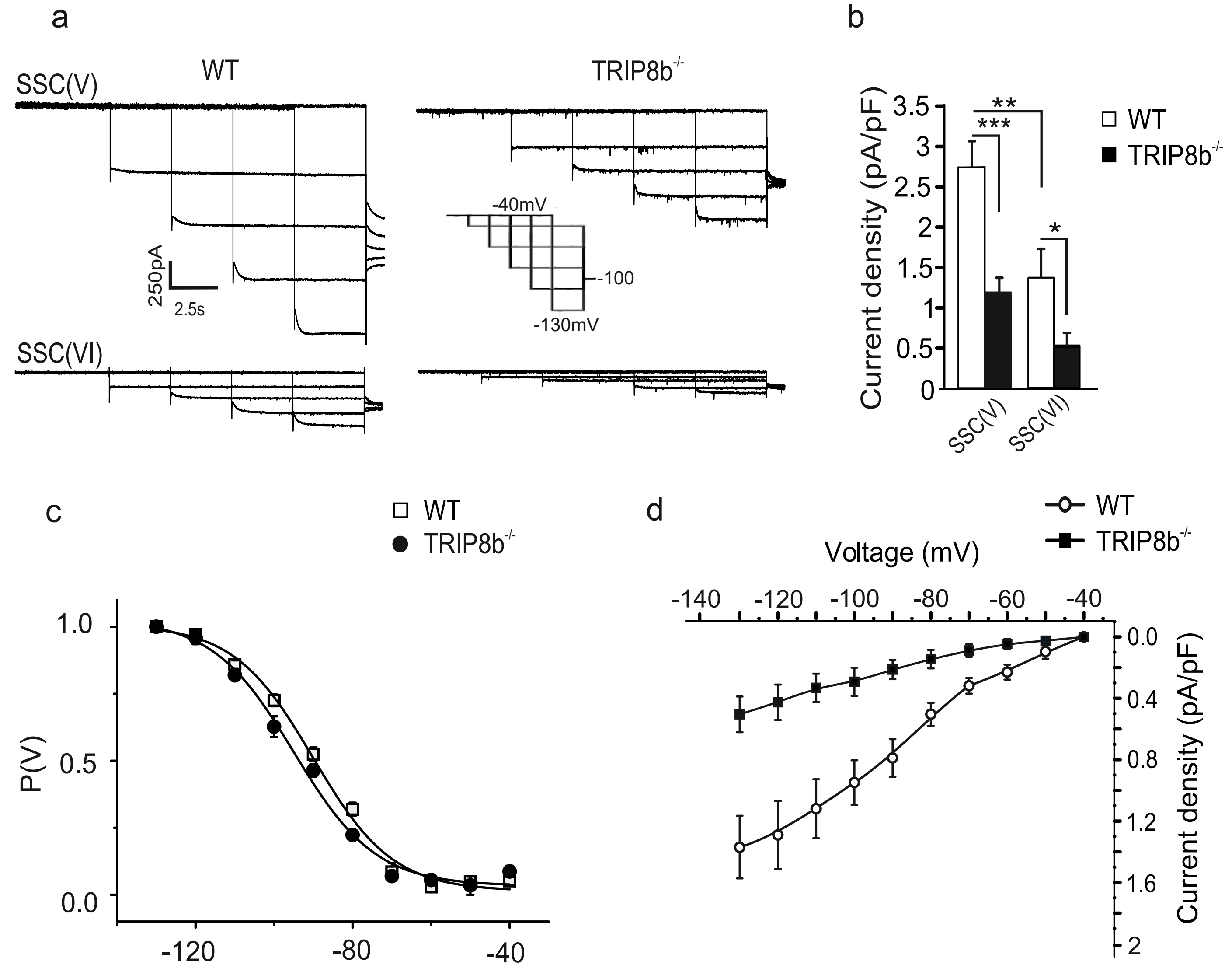

Supplemental Fig.7

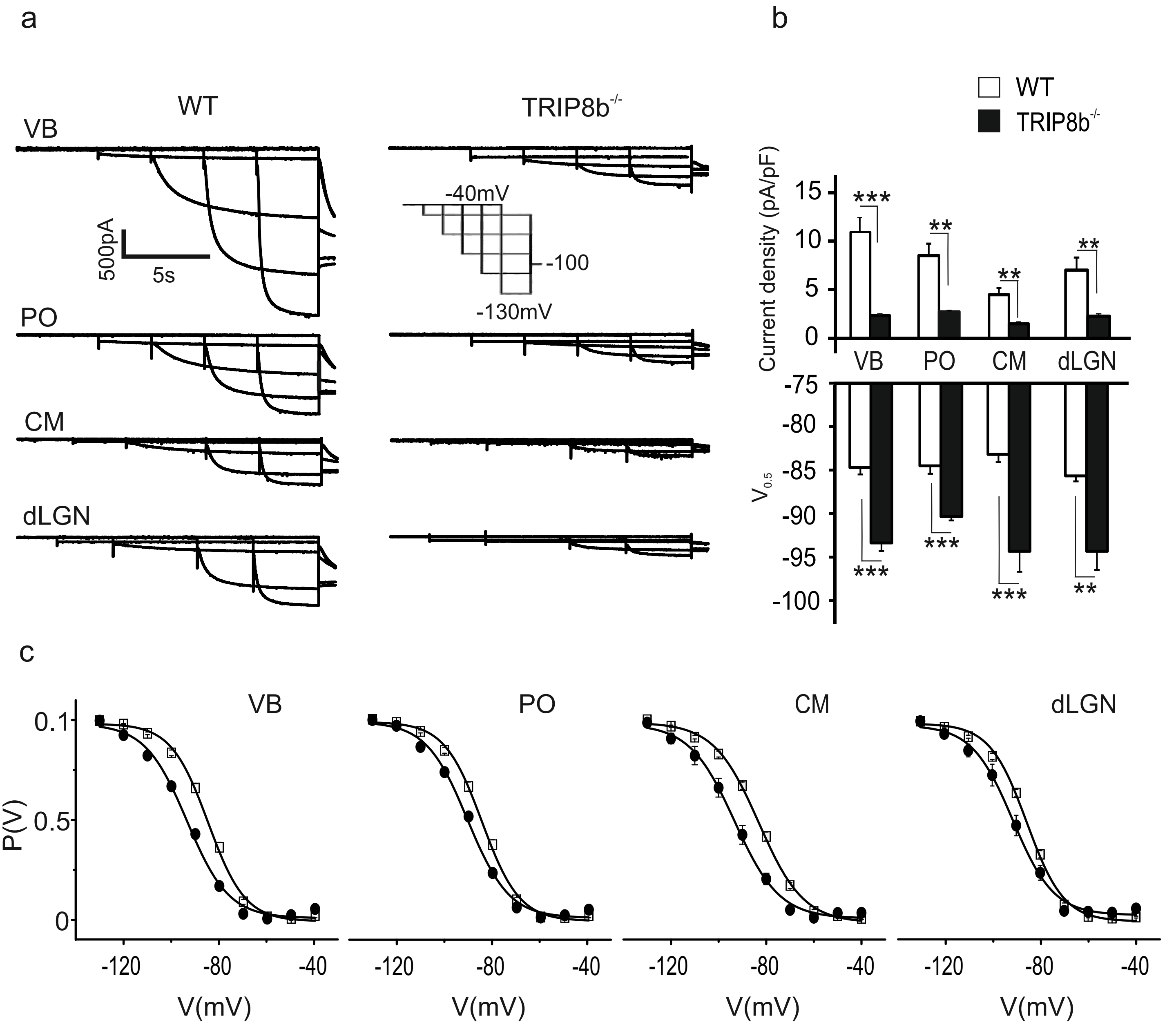

Supplemental Fig.8

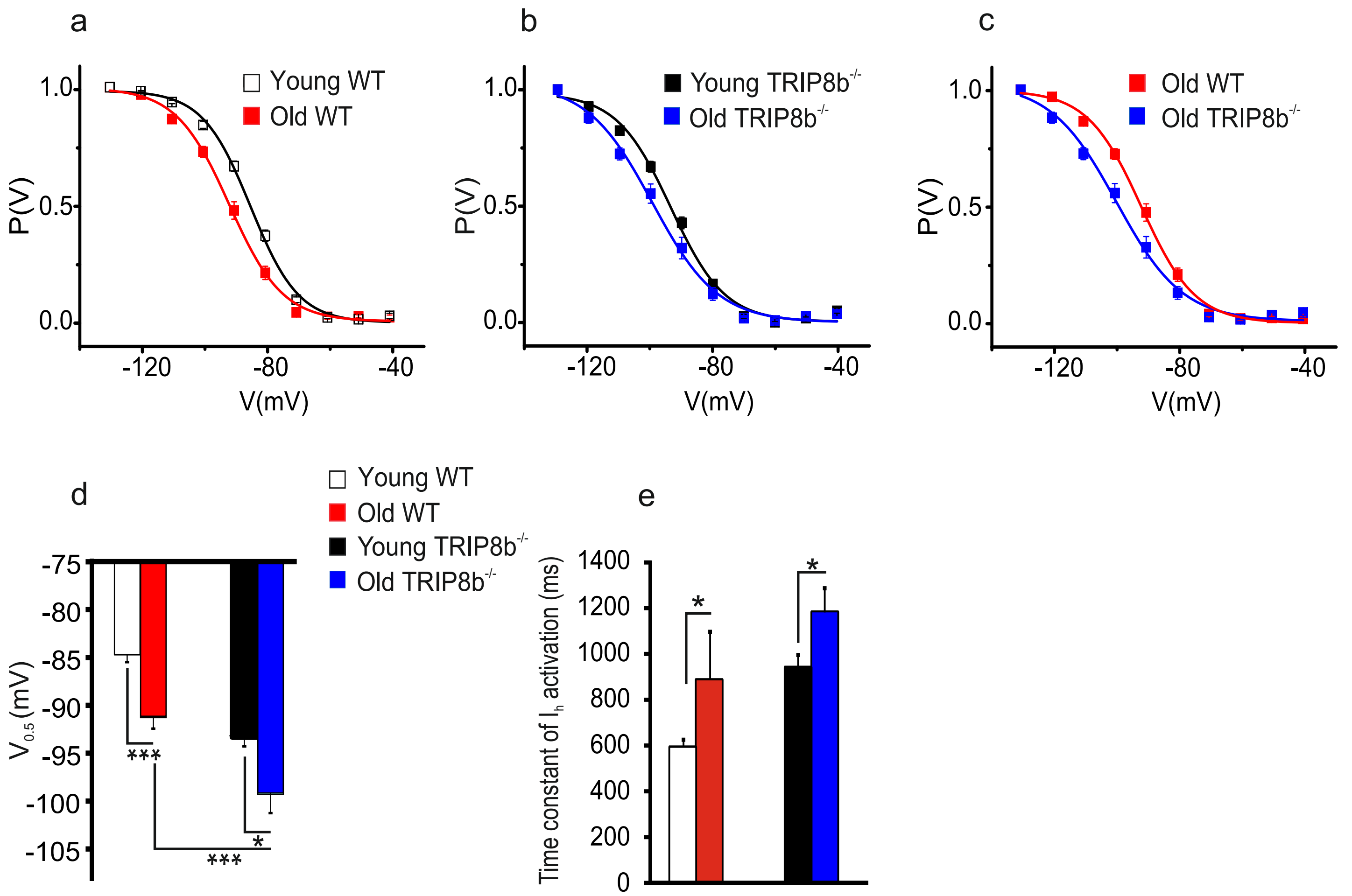

Supplemental Fig.9

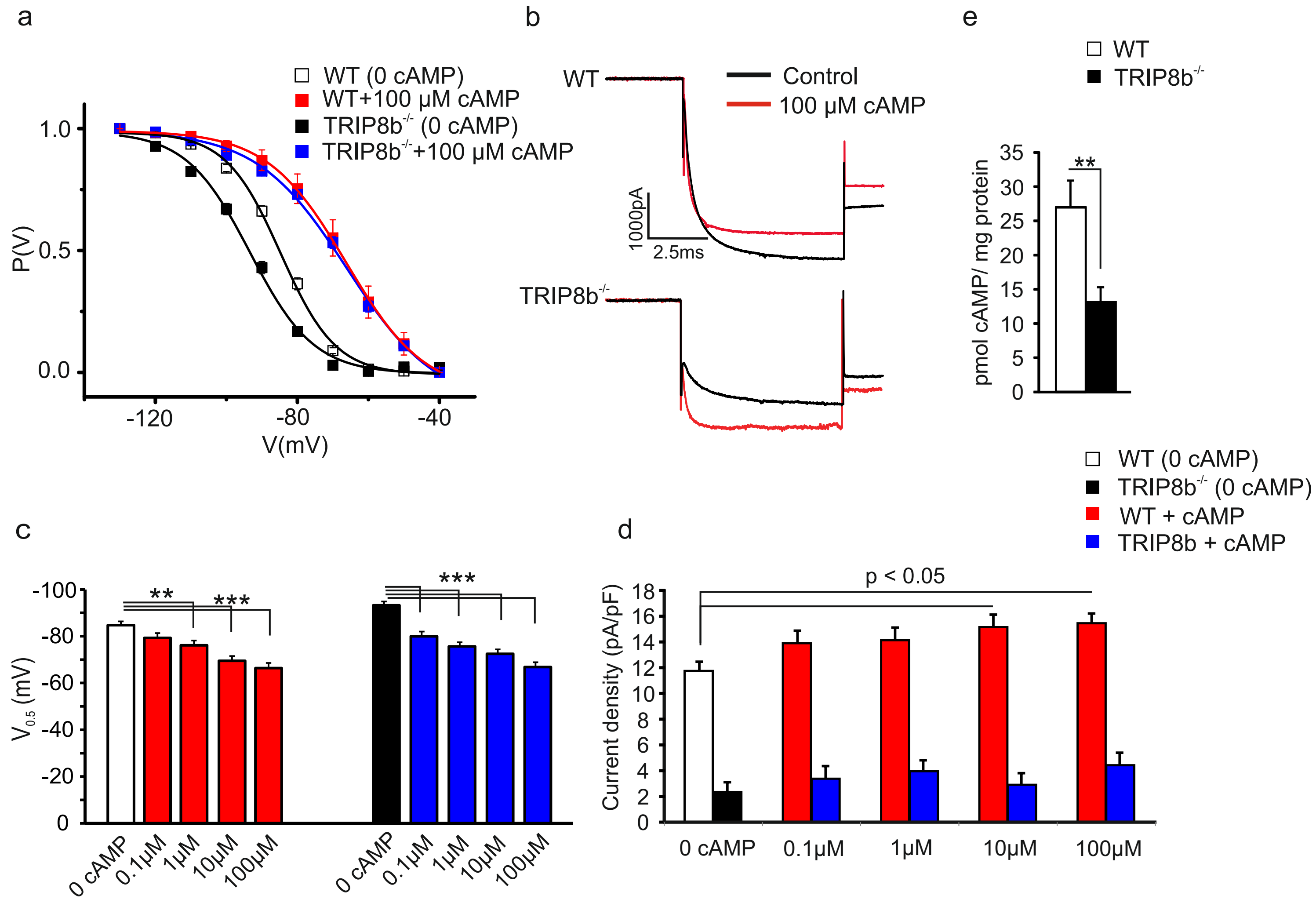

Supplemental Fig.10

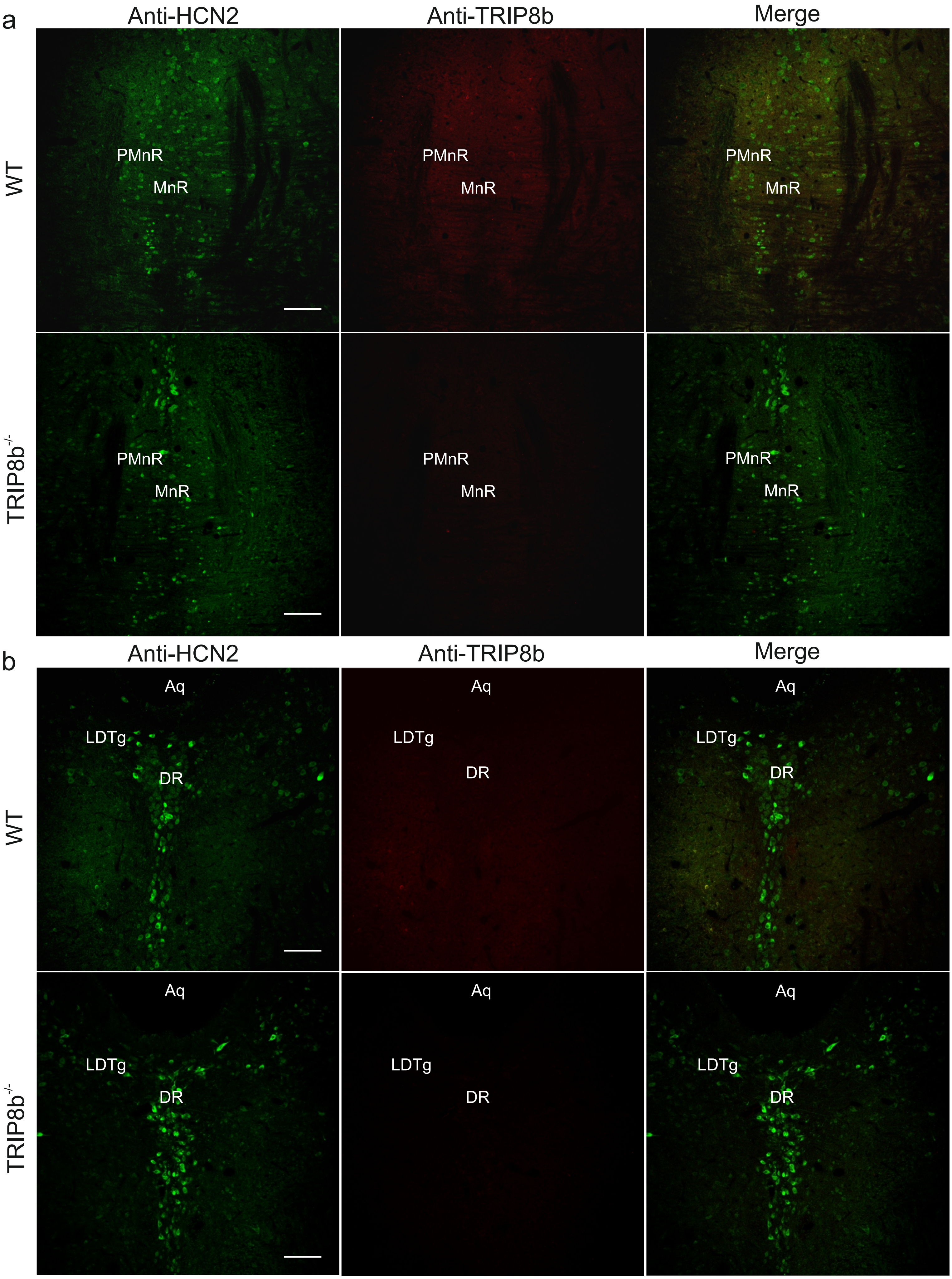

Supplemental Fig.11

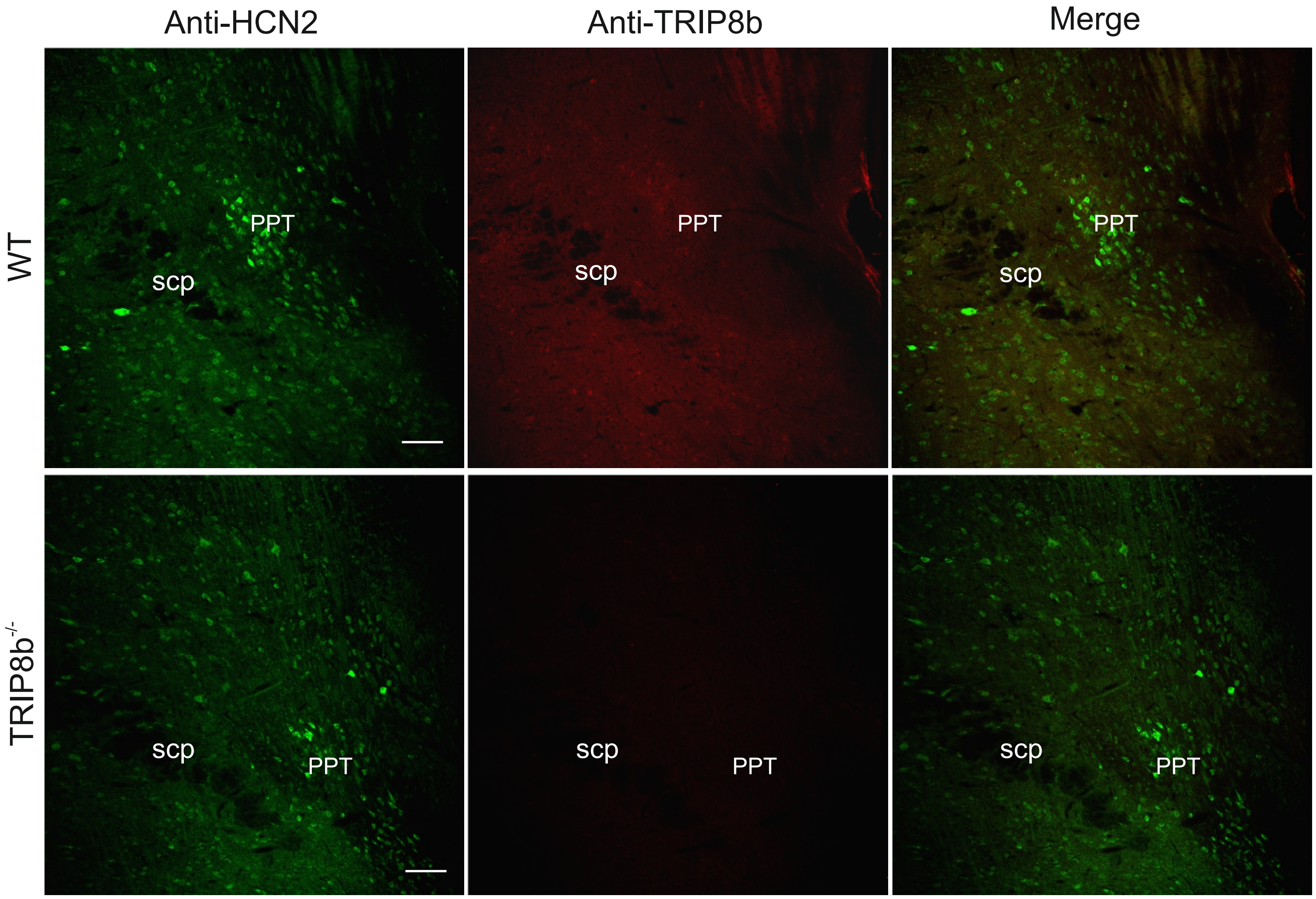

Supplemental Figures

Supplemental Fig. 1

TRIP8b does not change *hcn* channels gene expression pattern but regulates HCN protein expression in the thalamocortical system. The mRNA levels of *hcn 1 - 4* was quantified using qPCR on samples collected from the cortex and thalamus of WT and TRIP8b$^{-/-}$ mice. **a** Bar graphs comparing the expression level of *hcn1-4* genes. Data are presented as fold changes in mRNA expression level ($2^{-\Delta\Delta Ct}$) of *hcn 1-4* in TRIP8b$^{-/-}$ mice (n=4, P 25) compared to the WT (n=5, P 25). No difference in gene expression was detected for any of the four *hcn* genes in the somatosensory cortex (SSC), the posterior thalamic nucleus (PO), the ventral-basal complex (VB), the dorsal-lateral geniculate nucleus. For clarity the standard errors are deleted. **b** Immunohistochemical staining of HCN 1-2 channel subunits in layer V and VI $_{a\text{-}b}$ of the SSC of WT and TRIP8b$^{-/-}$ mice. Brain coronal sections (40 µm) from WT and TRIP8b$^{-/-}$ mice were stained with antibodies against HCN1 (rb-anti-HCN1, 1 : 200, depicted in red), HCN2 (rb-anti-HCN2, 1 : 200, depicted in red) and TRIP8b (ms-anti-TRIP8b; 1:50, depicted in green). In contrast to pyramidal neurons of layer V, a less dendritic expression of TRIP8b was detected in pyramidal neurons of layer VI $_{a\text{-}b}$ in WT mice. Here, TRIP8b was mainly expressed in somata and strongly overlapped with HCN1 and HCN2 subunits. Knockout of TRIP8b reduced the protein expression of the HCN1 and HCN2 subunits in layer VI $_{a\text{-}b}$. Scale bars indicate 100 µm. CC shows corpus callosum.

Supplemental Fig. 2

Downregulation of HCN2 and HCN4 subunits in dLGN of TRIP8b$^{-/-}$ mice. Brain coronal sections (40 µm) from WT and TRIP8b$^{-/-}$ mice were stained with antibodies against HCN2, HCN4 (rb-anti-HCN2, 1 : 200 and rb-anti-HCN4, 1 : 200, depicted in red) and TRIP8b (ms-anti-TRIP8b; 1:50, depicted in green). Compared to the HCN4 subunit which has a dendritic expression pattern, HCN2 is mostly expressed in the somata of thalamic relay neurons. Note the lower expression of HCN2 and HCN4 subunits in TRIP8b$^{-/-}$ dorsal-lateral geniculate nucleus of the thalamus (dLGN). “hip” indicates the location of hippocampus in the slices. Scale bars indicate 50 µm.

Supplemental Fig. 3

Downregulation of HCN2 and HCN4 subunits in PO nucleus of TRIP8b$^{-/-}$ mice. Brain coronal sections (40 µm) from WT and TRIP8b$^{-/-}$ mice were stained with antibodies against HCN2 and HCN4 (rb-anti-HCN2, 1 : 200 and rb-anti-HCN4, 1 : 200, depicted in red) and TRIP8b (ms-anti-TRIP8b, 1 : 50, depicted in green). As

illustrated, TRIP8b$^{-/-}$ mice show a significant down regulation in HCN2 and HCN4 expression. Arrows indicate the location of the posterior thalamic nucleus (PO) in the slices. VB represents ventral-basal complex of the thalamus. Scale bars indicate 50 µm.

Supplemental Fig. 4

Downregulation of HCN2 and HCN4 subunits in CM nucleus of TRIP8b$^{-/-}$ mice. Brain coronal sections (40 µm) from WT and TRIP8b$^{-/-}$ mice were stained with antibodies against HCN2 and HCN4 (rb-anti-HCN2, 1 : 200 and rb-anti-HCN4, 1 : 200, depicted in red) and TRIP8b (ms-anti-TRIP8b, 1 : 50, depicted in green) in central-medial thalamic nucleus (CM) of WT and TRIP8b$^{-/-}$ mice. Arrow head indicates the location of CM. Note the lower expression of HCN2 and HCN4 subunits in CM of TRIP8b$^{-/-}$ compared to WT mice. PV represents paraventricular thalamic nucleus. Scale bars indicate 50 µm.

Supplemental Fig.5

TRIP8b is not expressed in GABAergic thalamic neurons and local circuit interneurons. Staining of the brain coronal sections (40µm) from GAD67/GFP knock-in mice with antibody against TRIP8b (mouse-anti-TRIP8b; 1:50, depicted in red). In these Knock-in mice, the GABAergic neurons are labeled by expression of a green fluorescent protein (GFP) under control of the glutamate decarboxylase (GAD67/Gad1) promoter. As illustrated, TRIP8b is neither expressed in GABAergic thalamic neurons of the reticular thalamic nucleus (nRT, **a**) nor in local circuit interneurons of the dorsal-lateral geniculate nucleus (dLGN, **b**) and somatosensory cortex (SSC, **b**).VB represents the anatomical location of ventral-basal thalamic nucleus. Scale bar indicates 50 µm.

Supplemental Fig. 6

Influence of TRIP8b on I_h in cortical pyramidal neurons. a Representative traces of I_h recorded under voltage-clamp conditions from pyramidal neurons in layer V and VI of the somatosensory cortex. Only five hyperpolarizing steps from a series of 10 are shown. The last step shown is the one to -130 mV. **b** Bar graph illustrating the reduction of I_h current density in neurons of TRIP8b$^{-/-}$ mice compared to WT in layer V (n=9/8 cells, ANOVA, *** p<0.001) and layer VI (n=6/6 cells, ANOVA, * indicates p<0.05). **c** Mean steady-state I_h activation curve of pyramidal neurons in layer V, showing the negative shift in $V_{0.5}$ for neurons from TRIP8b$^{-/-}$ mice compared to WT (n=9/8 cells, Student's t-tests, p<0.05). **d** The I/V-curves show the reduction in I_h

current density in pyramidal neurons (layer VI) from TRIP8b$^{-/-}$ compared to WT mice (n=6/6 cells, ANOVA, $p<0.05$).

Supplemental Fig.7

TRIP8b regulates I_h density and voltage-dependent activation in thalamic relay cells. a Representative traces of I_h recorded under voltage-clamp conditions in different thalamic nuclei of WT and TRIP8b$^{-/-}$ mice. I_h current was measured by hyperpolarizing steps of -10 mV increments from a holding potential of -40 to -130 mV. To yield the steady-state activation curve of I_h, the fraction of open channels, p(V), was calculated by normalizing the mean tail current amplitudes in response to an additional step of 1000 ms to -100 mV and plotted against the membrane voltage (mV). Only five hyperpolarizing steps from a series of 10 are shown. VB, PO, CM and dLGN stand for the ventral-basal complex, posterior thalamic nucleus, central-medial thalamic nucleus and dorsal-lateral geniculate nucleus, respectively. As shown in **a** and upper panel **b**, the absence of TRIP8b resulted in a significant downregulation of the I_h current (Student's T-test, *, **, *** indicate $p<0.05$, $p<0.01$, and $p<0.001$, respectively). In addition, as illustrated in **b** (lower panel), the absence of TRIP8b induced a significant negative shift in the half-maximal activation of I_h ($V_{0.5}$) in TRIP8b$^{-/-}$ relay cells. **c** Representative mean steady-state activation curves of I_h in TRIP8b$^{-/-}$ (filled circles) and WT (open squares) relay cells of different thalamic nuclei.

Supplemental Fig.8

Age-dependent changes in the voltage-gating of HCN channels in thalamic relay neurons. a, b and c Graphs showing the mean steady–state activation curves of I_h at different postnatal ages (p15-30 (young) vs. p120 (old)) in thalamic relay neurons of the ventral-basal complex (VB). **d** Bar graph showing an age-dependent shift to negative potentials in the voltage-dependent activation ($V_{0.5}$) of I_h in both TRIP8b$^{-/-}$ and WT mice. **e** Bar graph demonstrating the slower time constant of I_h activation in older animals in both groups. (Student's t-test, *, *** indicate $p<0.05$ and $p<0.001$).

Supplemental Fig.9

Modulation of I_h in thalamic relay neurons by cAMP. a Mean steady-state activation curves of I_h ($V_{0.5}$) in TRIP8b$^{-/-}$ and WT relay neurons of the ventral-basal complex (VB) in the absence and presence of 100 µM 8-Br-cAMP (cAMP). In both TRIP8b$^{-/-}$ and WT TC cells, intracellular application of 8-Br-cAMP shifts the

voltage-dependent activation of I_h to more depolarized potentials. **b** Representative I_h current traces recorded under voltage-clamp at the step to -130 mV; control conditions (0 8-Br-cAMP, black) and after application of 100 µM 8-Br-cAMP (red), showing the faster time constant of I_h activation in the presence of cAMP for both WT and TRIP8b$^{-/-}$ VB TC neurons. **c** Graphs comparing the voltage-dependent activation of I_h in the presence of different concentrations of 8-Br-cAMP in WT and TRIP8b$^{-/-}$ VB TC neurons. TRIP8b$^{-/-}$ TC cells showed a significantly (One-way ANOVA, ** and *** indicate $p<0.01$ and $p<0.001$) higher sensitivity to 8-Br-cAMP compared to WT. **d** Bar graphs demonstrating that the cAMP-dependent increase in I_h current density only happens in the presence of TRIP8b. **e** Bar graph comparing the basal cAMP levels in WT and TRIP8b$^{-/-}$ mice. Tissue samples were obtained from 3-month-old male mice (n=3/3 animals and total of 18 samples). Quantification of cAMP concentration (pmol cAMP/mg protein) revealed a significantly (Student's t-test, $p<0.01$) lower cAMP level in TRIP8b$^{-/-}$ compared to WT mice.

Supplemental Fig.10-11

TRIP8b does not control the HCN2 channel protein expression in the brainstem. Brain coronal sections (40 µm) from WT and TRIP8b$^{-/-}$ mice were stained with antibodies against HCN2 (rb-anti-HCN2, 1 : 200, depicted in green) and TRIP8b (ms-anti-TRIP8b; 1:50, depicted in red). Compared to the thalamus and cortex (see Fig.3 and Supplemental Fig. 1-5), the overall expression of TRIP8b protein in the brainstem (**a-b** upper panels) of the WT mice is noticeably lower. In addition, lack of TRIP8b does not affect the HCN2 protein expression in this part of the brain. Aq, cerebral aqueduct; DR, dorsal raphe; LDTg, later-dorsal tegmental nucleus; PMnR, paramedian raphe nucleus; MnR, medial raphe nucleus; PPT, Pedunculopontine nucleus; scp, superior cerebellar peduncle. Scale bars indicate 100 µm.
